# Taxonomic and Phylogenetic Studies of Saprobic Fungi Associated with *Mangifera indica* in Yunnan, China

**DOI:** 10.3390/jof9060680

**Published:** 2023-06-16

**Authors:** Er-Fu Yang, Dong-Qin Dai, Jayarama D. Bhat, Turki M. Dawoud, Itthayakorn Promputtha, Nimal Adikaram, Steven L. Stephenson, Samantha C. Karunarathna, Saowaluck Tibpromma

**Affiliations:** 1 Center for Yunnan Plateau Biological Resources Protection and Utilization, College of Biological Resource and Food Engineering, Qujing Normal University, Qujing 655011, China; erfu20170431@gmail.com (E.-F.Y.); cicidaidongqin@gmail.com (D.-Q.D.); 2 Department of Biology, Faculty of Science, Chiang Mai University, Chiang Mai 50200, Thailand; itthayakorn.p@cmu.ac.th; 3 Master of Science Program in Applied Microbiology (International Program), Faculty of Science, Chiang Mai University, Chiang Mai 50200, Thailand; 4 Department of Botany and Microbiology, College of Science, King Saud University, P.O. Box 2455, Riyadh 11451, Saudi Arabia; bhatdj@gmail.com (J.D.B.); tdawoud@ksu.edu.sa (T.M.D.); 5 Biology Division, Vishnugupta Vishwavidyapeetam, Ashoke, Gokarna 581326, India; 6 National Institute of Fundamental Studies, Kandy 20000, Sri Lanka; n.k.b.adikaram@gmail.com; 7 Department of Biological Sciences, University of Arkansas, Fayetteville, AR 72701, USA; slsteph@uark.edu

**Keywords:** *Acremoniisimulans hongheensis*, *Chaenothecopsis hongheensis*, *Hilberina hongheensis*, mango, new records, new species

## Abstract

Fungi are a large and diverse group of microorganisms, and although the estimated number of species ranges between 2 and 11 million, only around 150,000 species have been described thus far. The investigation of plant-associated fungi is beneficial for estimating global fungal diversity, for ecosystem conservation, and for the continued development of industry and agriculture. Mango, one of the world’s five most economically important fruit crops, is grown in over 100 countries and has been demonstrated to have a great economical value. During surveys of mango-associated saprobic fungi in Yunnan (China), we discovered three new species (*Acremoniisimulans hongheensis*, *Chaenothecopsis hongheensis* and *Hilberina hongheensis*) and five new records. The phylogenetic analyses of multi-gene sequences (LSU, SSU, ITS, *rpb2*, *tef1*-*α* and *tub2*) coupled with morphological examinations were used to identify all the taxa.

## 1. Introduction

Fungi prosper in various environments and play an important role in decomposition and nutrient cycling because of their ability to degrade cellulose and lignin [1,2]. A robust estimate of global fungal diversity is 2 to 11 million; however, only about 150,000 species are acknowledged to date, and tropical to subtropical areas appear to have a particularly high diversity of undiscovered fungi [3,4,5]. Fungi are distributed almost everywhere, including soil, marine water, freshwater, air, plants, animals and humans [2]. Hyde et al. [1] indicated that fungi have an enormous economic potential and listed 50 different ways that we can exploit the fungi industrially including their use in strategies against human diseases; as biocontrol agents against pests and bacterial or other fungal pathogens on plants; to enhance plant growth; for improving food and beverages; and as various commodities.

Mango is native to south Asia, India and the Malay Archipelago [6,7]. Subsequently, mango was introduced to over 100 countries, with more than 1000 varieties developed worldwide, and has always been considered an important cash crop in tropical to subtropical regions [8]. In China, the commercial cultivation of mango begun between the 1960s and 1980s and then grew rapidly, leading to China becoming the third-largest mango-producing country in the world, with 294,326 hectares of plants and 2,414,800 tons of harvested fruits in 2018 [9]. Yunnan, one of the mango-growing provinces in China, supplying 20% of the total mangoes produced nationwide, generated an economic value of CNY 1.8 billion in 2018 [10]. The main production areas in the Yunnan Province are Baoshan, Honghe, Huaping, Jinghong, Simao and Yuanjiang, and the main varieties are Keitt, Guifei, Sannian, Nang Klangwan and Jin Hwang [9].

While the investigation of plant-associated fungi contributes to the estimation of global fungal diversity, it also benefits global ecosystem conservation and industrial and agricultural development [11]. Taïbi et al. [12] mentioned that a total of 866 genera of bacteria and fungi are associated with mango, of which over 2000 species belonging to 304 genera are fungi. However, most mango-associated fungi reported earlier lack morphological details and phylogenetic supports due to limitations in the use of molecular techniques and good laboratory conditions. More recently, many mango-associated fungi have been reported along with convictive phylogenetic analysis and morphological characteristics. Recently, Tennakoon et al. [13] introduced the new species *Pseudolophiostoma mangiferae* and a new record of *Neovaginatispora fuckelii* from mango. Guo et al. [14] reported seven species of *Fusarium* from leaf spots of mango. Tamakaew et al. [15] established a new species of *Cercosporoid* fungus associated with mango in Thailand. Yang et al. [16,17] reported 20 saprobic fungi that are associated with mango in China. Therefore, many more mango-associated fungi are likely to be found in the future. In this paper, three new species and five new records of mango-associated saprobic fungi are described and introduced based on morphological comparisons and multi-gene phylogenetic analyses.

## 2. Materials and Methods

### 2.1. Sampling and Isolation

The dead and decaying woody specimens of mango were collected from rural areas of Baoshan and Honghe (Yunnan, China). The samples consisting of decayed branches, barks, and twigs (Keitt and Guifei varieties) of mango were collected and taken to the mycology laboratory. A stereo microscope (Olympus SZ61; Olympus corporation, Tokyo, Japan) was used for observing the fungal fruiting bodies on plant materials, while a digital Canon camera (EOS 600D, Canon Inc., Tokyo, Japan) fitted on to a compound microscope (Nikon ECLIPSE Ni, Nikon., Tokyo, Japan) was used to capture micro-morphological characteristics. The sizes of the main structures of fungi such as ascomata, ascomata wall, paraphyses, asci/conidiogenous cells and ascospores/conidia were measured by the Tarosoft(R) Image Frame Work program (IFW). The color photos were combined in Adobe Photoshop CS3 Extended v. 10.0 (Adobe**^®^**, San Jose, CA, USA) to prepare complete photo plates.

Pure cultures were obtained by the single spore isolation method [18]. The spores/conidial masses were collected with a sterilized vaccination needle and gently dispersed on sterile water droplets on a micro slide. The spore suspension was transferred onto potato dextrose agar (PDA) using a micropipette and kept at 27 °C for one to two nights for germination. The single germinated spores were aseptically placed on new PDA plates, and incubated at 27 °C. The growth rate and colony characteristics of the fungal isolate were recorded after 10 days. Dried plant specimens with fungal fruiting bodies were deposited in the herbarium of the Kunming Institute of Botany Academia Sinica (HKAS), while fungal culture in tubes were deposited in the Kunming Institute of Botany Culture Collection (KUMCC). Fungal name numbers of novel species were registered as per the instruction (https://nmdc.cn/fungalnames/, accessed on 3 February 2023) [19].

### 2.2. DNA Extraction, PCR and Sequencing

After the culture grew for half a month, the fresh mycelium (50–100 mg) was picked up by a sterilized needle and stored in 1.5 mL centrifugal tubes for DNA extraction, for those fungal species from which we could not obtain pure cultures, approximately 5–10 fruiting bodies were carefully collected in centrifugal tubes for DNA extraction. The genomic DNA was extracted following the user instruction book of the Biospin Fungus Genomic DNA Extraction Kit-BSC14S1 (BioFlux^®^, Beijing, China). A part of the extracted DNA was used as template for polymerase chain reaction (PCR), while the other part was used for long-term storage at −20 °C. The 25 µL PCR mixture contained double-distilled water (8.5 µL), 2 × Power Taq PCR MasterMix (12.5 µL), DNA template (2 µL) and 2 µL of forward and reverse primers (10 pmol) (Table 1) [16].

The genes ITS, LSU, *rpb2*, SSU, *tef1-α* and *tub2* were amplified, and the different primers used in this study are shown in Table 1. The purification and sequencing of PCR products were performed at Beijing Bio Teke Corporation.

### 2.3. Phylogenetic Analyses

Geneious (Restricted) 9.1.2 was used to assemble forward and reverse sequences (https://www.geneious.com, accessed on 12 May 2023), and then those combined sequences were searched in BLASTn of GenBank (http://blast.ncbi.nlm.nih.gov/, accessed on 12 May 2022) to screen relatively highly similar genera/taxa for the phylogenetic analyses (Tables S1–S8). Sequence alignments were made at the MAFFT online server version (www.ebi.ac.uk/Tools/mafft, accessed on 12 May 2022) [25] and slightly modified in BioEdit 7.2.3 [26] whenever necessary. Uninformative and unclear regions of sequences were removed by trimAL v1.2 (http://trimal.cgenomics.org, accessed on 12 May 2022), while alignments of different genes were combined in BioEdit. The online program Alignment Transformation Environment (ALTER) was used to convert Fasta files to PHYLIP (for ML) and NEXUS (for BI) formats [27]. The Maximum Likelihood analysis (ML) was run in the CIPRES Science Gateway v.3.3 (http://www.phylo.org/portal2, accessed on 12 May 2022) [28] by selecting RAxML-HPC2 on XSEDE (8.2.12) [29] and 1000 bootstrap iterations in the GTRGAMMA substitution model. The Bayesian analysis was conducted in MrBayes on XSEDE (3.2.7a) via the CIPRES Science Gateway V.3.3 web server [29,30]. Bayesian posterior probabilities (BYPP) [31,32] were assessed by Markov Chain Monte Carlo sampling (MCMC), using the best models of evolution determined by MrModeltest v. 2.3 [33] and PAUP v. 4.0b10 [34]. The GTR+I+G evolution model was also run in the BI analysis. Six simultaneous Markov chains were simultaneously subjected to Bayesian analysis for 1,000,000 to 10,000,000 generations, depending on various parameters; samples of the fungi and trees were taken, printed and produced every 1000th generation. Multi-gene phylogenetic trees were opened and checked using FigTree v1.4.0 [35], and the final trees were edited in Microsoft PowerPoint by inserting statistical supports from ML and BI.

## 3. Results

### Taxonomic and Phylogenetic Results

Sordariomycetes O.E. Erikss & Winka 1997

Glomerellales Chadef. ex Réblová, W. Gams & Seifert, Stud. Mycol. 68: 170 (2011)

Plectosphaerellaceae W. Gams, Summerb & Zare 2007

*Acremoniisimulans* Tibpromma & K.D. Hyde, Fungal Diversity 93: 88 (2018)

Index Fungorum number: IF555329

Type species: *Acremoniisimulans thailandensis* Tibpromma & K.D. Hyde, Fungal Diversity 93: 89 (2018)

**Figure 1:**
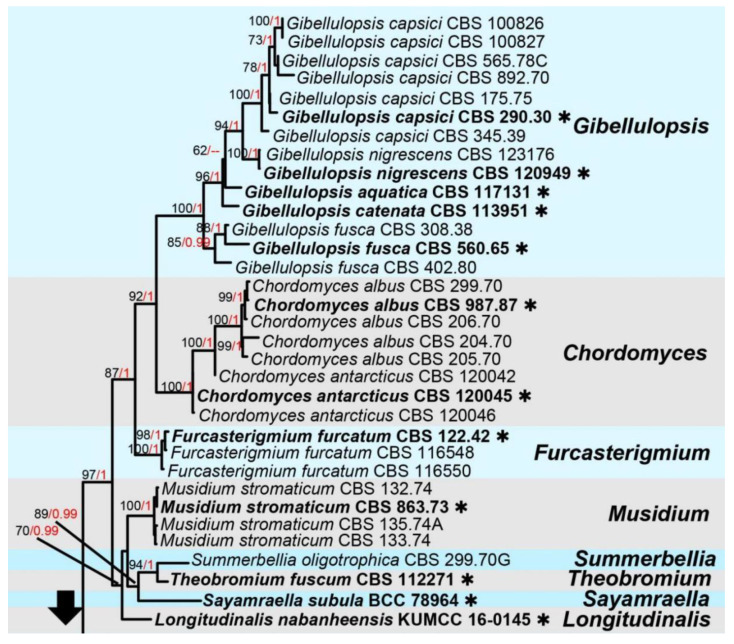

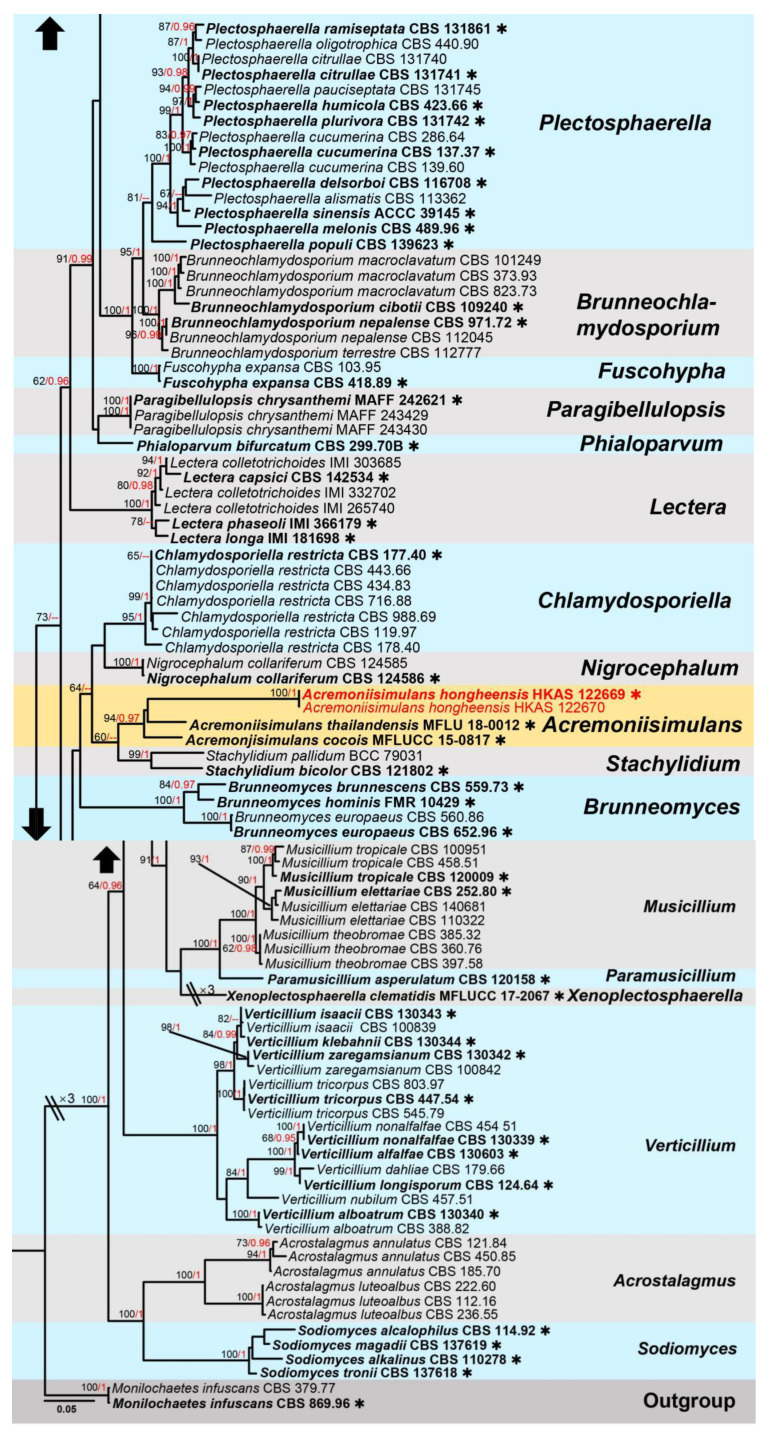
The phylogram based on a Maximum Likelihood analysis of combined LSU, ITS, *tef1*-*α* and *rpb2* sequence datasets. The analyzed gene contains 124 fungal strains and 2903 total characters including gaps (LSU: 1–833 bp, ITS: 834–1368 bp, *tef1*-*α*: 1369–2160 bp, *rpb2*: 2161–2903 bp). The tree topology of the ML resembles BI. The matrix had distinct alignment patterns, with the final ML optimization likelihood value of −32,895.306654 (ln). All free model parameters were estimated by the RAxML model, with 1112 distinct alignment patterns and 9.75% of undetermined characters or gaps. Estimated base frequencies were as follows: A = 0.225219, C = 0.295585, G = 0.280821 and T = 0.198375, with substitution rates AC = 0.822329, AG = 2.159486, AT = 1.120296, CG = 0.716819, CT = 5.475391 and GT = 1.000000. The gamma distribution shape parameter alpha = 0.656730, and the Tree-Length = 3.625234. The final average standard deviation of split frequencies at the end of total MCMC generations was calculated as 0.009897 in BI analysis. The type strains are denoted in bold with the symbol “✱” at the ends, and newly introduced species in this study are denoted in red. The nodes provide bootstrap values of at least 60% (ML, left) and Bayesian posterior probabilities of at least 0.95 (BI, right); hyphens (-) signify values that are less than 60% in ML and less than 0.95 in BI. The bluish and pale brown backgrounds were used to distinguish different genera in Plectosphaerellaceae, while the yellow background indicates the genus *Acremoniisimulans*.

Notes: Genus *Acremoniisimulans* was established with the type species *A. thailandensis,* found associated with *Pandanus* sp. in Thailand, wherein only the asexual morph of this species was available [36]. Recently, Konta et al. [37] introduced a new species in this genus, a sexual morph *Acremoniisimulans cocois,* on dead petioles of *Cocos nucifera* (Arecaceae) in Thailand. To date, only above two species are accommodated in *Acremoniisimulans* [37]. The sexual morph of *Acremoniisimulans* was described as producing four or eight biseriate, oblong to broadly oblong, straight or curved, hyaline, one-celled, guttulate ascospores in each oblong to clavate, and pedicellate ascus; *Acremoniisimulans* asexually described by having solitary, hyaline or subhyaline to pale brown, oval, aseptate, slimy, conidia formed on macronematous, mononematous, scattered, smooth, and thick-walled conidiophores [36,37]. The multi-gene phylogenetic placements of *Acremoniisimulans* sp. and other genera in Plectosphaerellaceae are shown in Figure 1.

**Figure 2:**
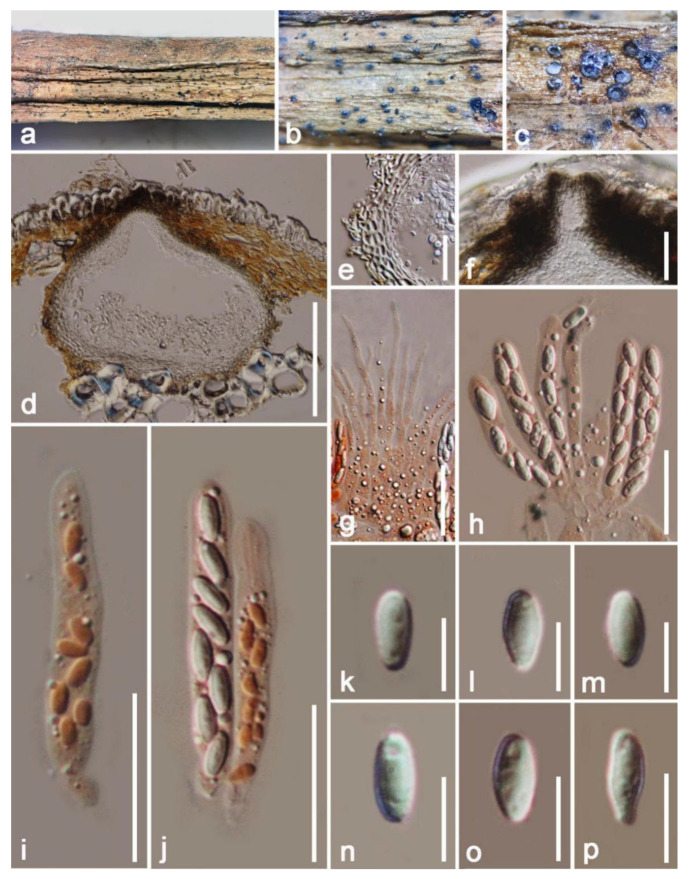
*Acremoniisimulans hongheensis* (HKAS 122669, holotype). (**a**,**b**) Ascomata immersed on the plant host; (**c**) horizontal section of ascomata; (**d**) vertical section of ascoma; (**e**) ascomata wall; (**f**) ostiole; (**g**) paraphyses stained by Congo red reagent; (**h**–**j**) mature and immature asci stained by Congo red reagent; (**k**–**p**) ascospores stained by Congo red reagent. Scale bars: (**d**) =100 μm; (**f**,**e**) = 50 μm; (**f**,**g**) = 30 μm; (**h**–**j**) = 20 μm; (**k**–**p**) = 5 μm.

*Acremoniisimulans hongheensis* E.F. Yang & Tibpromma, sp. nov. (Figure 2)

Fungal Name number: FN571274

Etymology: The name reflects the location, Honghe, from where the holotype was collected.

Holotype: HKAS 122669

*Saprobic* on dead twigs of *Mangifera indica*. Sexual morph: Visible as numerous black, raised dots beneath the epidermis of the host. *Ascomata* (excluding neck) 120–150 μm × 120–195 μm ($\bar{x}$ = 140 × 160 μm, n = 10) diam., immersed, globose to subglobose, exposed when horizontally sectioned through bark surface, scattered to gregarious, with a short ostiole. *Ostiole* 50–75 μm × 50–75 μm ($\bar{x}$ = 65 × 60 μm, n = 10) diam., central, cylindrical to narrowly canalled, with darkly pigmented cells in the neck. *Ascomata wall* 15–25 μm ($\bar{x}$ = 19 μm, n = 20) wide, multilayered, with inner layers comprising hyaline, long and narrow cells of *textura prismatica*, outer layers formed with thick-walled cells of *textura angularis*. *Hamathecium* composed of 2–5 μm ($\bar{x}$ = 4 μm, n = 20) wide, filamentous, dense, cylindrical, septate, unbranched, straight to slightly curved, paraphyses formed from a gelatinous matrix. *Asci* 40–50 × 5–7 μm ($\bar{x}$ = 46 × 6 μm, n = 10), eight spored, bitunicate, oblong, cylindrical, hyaline, short pedicellate, apically rounded, with a vague apical chamber. *Ascospores* 5–8 × 2–4 μm ($\bar{x}$ = 6.5 × 3 μm n = 20), uni- or bi-seriately overlapping, ellipsoidal, septate, obtuse at both ends, thick and smooth walled, without a gelatinous sheath or appendages. Asexual morph: Undetermined.

Material examined: China, Yunnan Province, Baoshan City, Longling County, on dead twigs of *Mangifera indica* (99°16′80″ E, 25°12′23″ N, Elevation: 800 m) 27 December 2019, E.F. Yang, erfu1 (Herb. HKAS 122669, holotype); isotype HKAS 122670. GenBank numbers: HKAS 122669 = ITS: OQ379005, LSU: OQ379416, SSU: OQ372921, *tef1*-*α*: OQ378995, *rpb2*: OQ378988; HKAS 122670 = ITS: OQ379006, LSU: OQ379417, SSU: OQ372922, *tef1*-*α*: OQ378996, *rpb2*: OQ378989.

Notes: Based on morphology, our isolate shares similar sexual morph with *Acremoniisimulans cocois* (Plectosphaerellaceae), by producing biseriate asci, oblong to ellipsoidal, hyaline, 1-celled, guttulate, thick and smooth-walled ascospores and without a gelatinous sheath or appendages [37]. In addition, the BLASTn results of LSU and SSU showed 97–99% similarity with the *Acremoniisimulans thailandensis* (MFLUCC 16-0372) and *A. cocois* (MFLUCC 15-0817). However, the BLASTn results of ITS and *tef*-*α* showed a low similarity (91%) with *Verticillium isaacii* (CBS100839), *V. tricorpus* (CBS100867) and *Chlamydosporiella restricta* (CBS 178.40, CBS177.40); in addition, the *rpb2* gene even has a similarity of only 79% with *Acremonium collariferum* (CBS124585). The multi-gene phylogenetic trees also indicated that *Acremoniisimulans hongheensis* strains separated from *A. cocois* and *A. thailandensis* (Figure 1). Therefore, *Acremoniisimulans hongheensis* is established as the third species in the genus *Acremoniisimulans* [37].

Dothideomycetes sensu O.E. Erikss & Winka

Tubeufiales Boonmee & K.D. Hyde, in Boonmee, Rossman, Liu, Li, Dai, Bhat, Gareth Jones, McKenzie, Xu & Hyde, Fungal Diversity 68(1): 245 (2014)

Tubeufiaceae M.E. Barr, Mycologia 71(5): 948 (1979)

*Excipulariopsis* P.M. Kirk & Spooner, in Spooner & Kirk, Trans. Br. mycol. Soc. 78(2): 251 (1982)

Index Fungorum Registration Identifier: IF8228

Type species: *Excipulariopsis narsapurensis* (Subram.) Spooner & P.M. Kirk 1982

**Figure 3:**
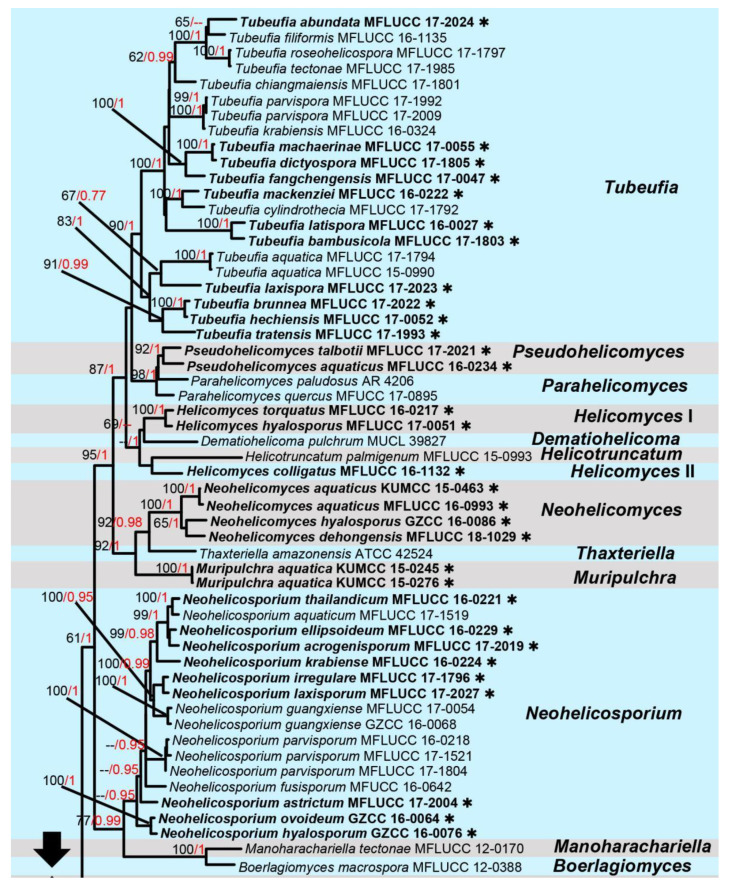

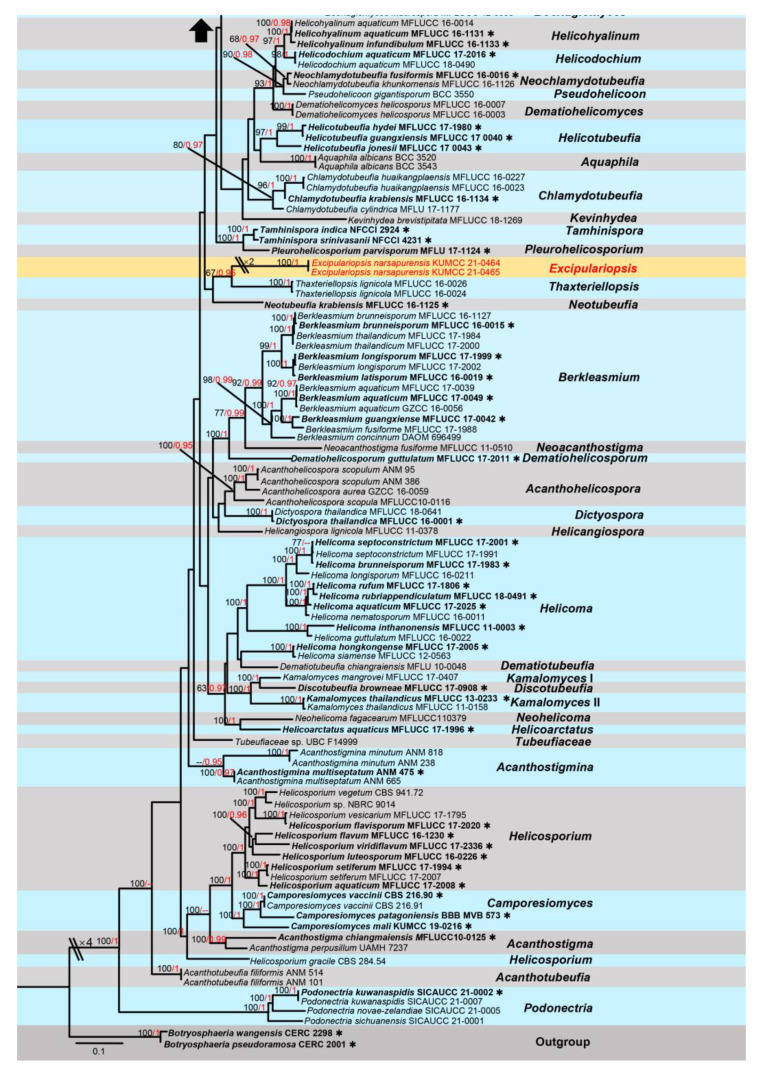
The phylogram based on a Maximum Likelihood analysis of combined LSU, *tef1*-*α*, *rpb2* and ITS sequence datasets. The analyzed gene contains 154 fungal strains and 3832 total characters including gaps (LSU: 1–853 bp, *tef1*-*α*: 854–1769 bp, *rpb2*: 1770–3228 bp, ITS: 2819–3438 bp). The tree topology of the ML resembles BI. The matrix had distinct alignment patterns, with the final ML optimization likelihood value of −59,582.416081 (ln). All free model parameters were estimated by RAxML model, with 1781 distinct alignment patterns and 21.90% of undetermined characters or gaps. Estimated base frequencies were as follows: A = 0.242692, C = 0.256407, G = 0.261351 and T = 0.239550, with substitution rates AC = 1.186006, AG = 5.169063, AT = 2.123954, CG = 0.808118, CT = 10.262422 and GT = 1.000000. The gamma distribution shape parameter alpha = 0.705889, and the Tree-Length = 10.099715. The final average standard deviation of split frequencies at the end of total MCMC generations was calculated as 0.009897 in BI analysis. The type strains are denoted in bold with the symbol “✱” at the ends, and newly introduced species in this study are denoted in red. The nodes provide bootstrap values of at least 60% (ML, left) and Bayesian posterior probabilities of at least 0.95 (BI, right); hyphens (-) signify values that are less than 60% in ML and less than 0.95 in BI. The bluish and pale brown backgrounds were used to distinguish different genera in Tubeufiaceae, while the yellow background indicates the genus *Excipulariopsis*.

Notes: The family Tubeufiaceae contains about 47 genera and more than 400 species [38]. The monotypic genus *Excipulariopsis* was established with *E. narsapurensis* as the type species [39], and the asexual morph has raised stroma, cylindrical, hyaline, holoblastic conidiogenous cells and fusiform, transversely multi-septate, brown, verruculose, acrogenous conidia with a truncate base [39,40]. However, molecular data for *Excipulariopsis narsapurensis* are unavailable, and therefore, the phylogenetic placement remained unsettled until this study. Multi-gene BLASTn (ITS, LSU, *tef1*-*α* and *rpb2*) searchers of our isolate remained relatively low in similarity with the strains of other genera. The BLASTn searches of ITS and *tef1*-*α* of our isolates (KUMCC 21-0464, 21-0465) showed a low similarity (85–87%) with *Chlamydotubeufia khunkornensis* (MFLUCC 10-0117), *Tubeufiaceae* sp. (MFLUCC 16-1129), *Helicosporium flavum* (MFLUCC 16-1230) and *Tubeufia tectonae* (MFLUCC 17-1985); the LSU gene has a similarity of 96% to *Parawiesneriomyces chiayiensis* (MFLUCC 20-0041) and *Thaxteriellopsis lignicola* (MFLUCC 16-0026); and the *rpb2* gene has a similarity of 80% to *Berkleasmium latisporum* (MFLUCC 16-0019) and *Helicoma multiseptatum* (GZCC 16-0080). The multi-genetic phylogenetic trees (ML and BI) formed a well-supported subclade close to genus *Thaxteriellopsis* with low statistical support (66% in ML, 0.99 in BI; Figure 3), but they were well separated from *Neotubeufia* and *Thaxteriellopsis.* Therefore, this study showed the phylogenetic placement of the genus *Excipulariopsis* for the first time (Figure 3).

**Figure 4:**
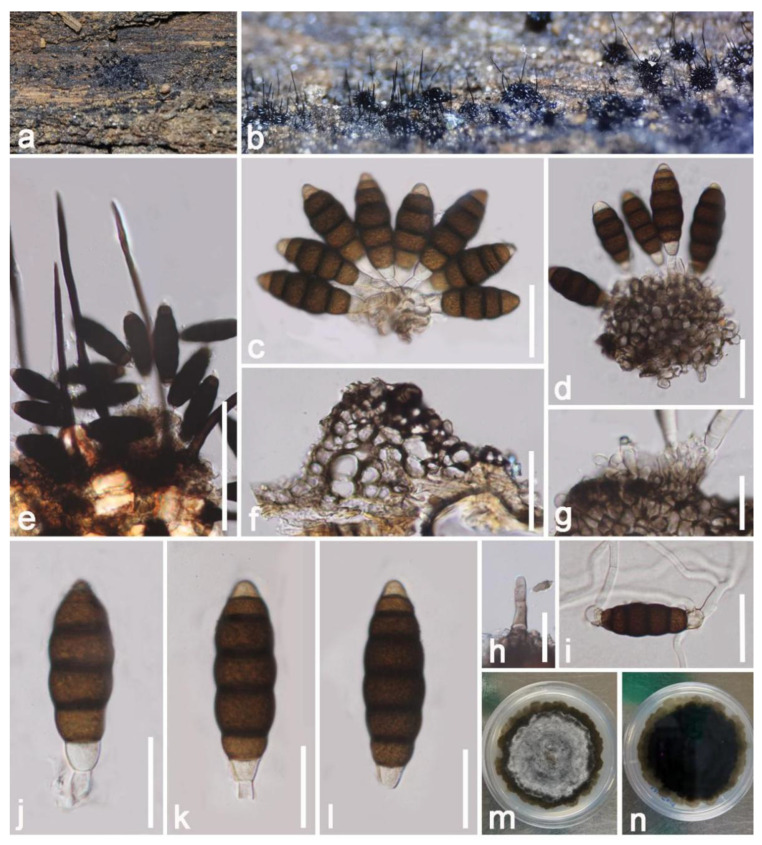
*Excipulariopsis narsapurensis* (HKAS 122680). (**a**,**b**) Colonies on the natural substrate; (**e**) colonies as observed by microscope; (**c**,**d**) conidia with conidiogenous cells; (**f**) a stroma; (**g**) conidiogenous cells; (**h**) a seta; (**j**–**l**) conidia; (**i**) germinated conidium; (**m**) colony from above in PDA; (**n**) colony from below in PDA. Scale bars: (**e**) = 100 µm; (**c**,**d**,**f**,**i**) = 30 µm; (**g**) = 30 µm; (**j**–**l**) = 20 µm.

*Excipulariopsis narsapurensis* (Subram.) Spooner & P.M. Kirk, Trans. Br. mycol. Soc. 78(2): 251 (1982) (Figure 4)

=*Excipularia narsapurensis* Subram., J. Indian bot. Soc. 35(1): 56 (1956)

Index Fungorum Registration Identifier: IF110673

*Saprobic* on a dead bark of *Mangifera indica*. Sexual morph: Unknown. Asexual morph: Hyphomycetous. *Colonies* gregarious, effuse, superficial, pulvinate, setiferous, sporodochial, recognized as shining black regions, distinctly scattered on the surface of the host, with erect, 7–10 μm-wide setae. *Stroma* composed of dark, thick, brown-walled stromatic cells. *Setae* erect, straight, septate, tubular, black, peripheral, smooth, with acute ends. *Conidiogenous cells* 12–20 × 4–6 μm ($\bar{x}$ = 16 × 5 μm), holoblastic, cylindrical, hyaline to lightly brown, smooth walled, straight to slightly flexuous, arise on stroma as a palisade layer. *Conidia* 40–55 × 9–30 μm ($\bar{x}$ = 48 × 19 μm n = 20), 4–7 septate, acrogenous, fusiform, markedly constricted at the septum, smooth walled, apically rounded at both ends, granulate, thick walled, brown to dark brown, hyaline to pale brown at both end cells, with thick and dark bands at septa, often carrying a small part of conidiogenous cell at the base.

Culture characteristics: Conidia produced germ tubes from both ends within 24 h at 27 °C, rapidly growing, colony up to 20–30 mm after one week; obverse: circular, flat or effuse, crenated at the edge, white to gray pigments at the center, with medium-level density; reverse: Brown to dark brown from outer to inner parts, pale brown at the margin, without pigments produced in PDA.

Substratum: On decaying wood with corticeaceous fungus [39,40]; *Cocos nucifera* [41], the bark of *Mangifera indica* (this study).

Distribution: Hawaii, USA [39,40]; Dapoli, India [41]; Yunnan Province, China (this study).

Material examined: China, Yunnan Province, Honghe, Menglong Village, on a dead and decaying bark of *Mangifera indica* (102°50′11″ E, 23°41′01″ N, 500 m), 22 December 2020, E.F. Yang, HHE018 (HKAS 122680), living culture, KUMCC 21-0464 = KUMCC 21-0465. Genbank number: KUMCC 21-0464 = ITS: OQ379007, LSU: OQ379418, *tef1*-*α*: OQ378997, *rpb2*: OQ378990;KUMCC 21-0465 = ITS: OQ379008, LSU: OQ379419, *tef1*-*α*: OQ378998, *rpb2*: OQ378991.

Notes: The asexual morphs of Tubeufiaceae are hyphomycetous, often with helicosporous, phragmosporous or sometimes chlamydosporous conidia [42,43]. In this study, our collection resembled *Excipulariopsis narsapurensis* (BISH 594584) in having effuse, superficial, sporodochial colonies, with cylindrical, hyaline, holoblastic conidiogenous cells and brown, phragmoseptate, acrogenous conidia (68–72 × 23–27 µm vs. 40–55 × 9–30 μm [40]. Multi-gene BLASTn (ITS, *tef1*-*α* and *rpb2*) searchers showed relatively low similarity (<90%) with strains of other genera (e.g., *Chamydotubeufia*, *Thaxteriellopsis*, *Tubefufia* and *Neoacanthostigma*). Based on multi-gene analyses of LSU, *tef1*-*α*, *rpb2* and ITS datasets, strains of *Excipulariopsis narsapurensis* are seen as clearly distinguishable from other generic species in the Tubeufiaceae. Therefore, in this study, with the phylogenetic placements and molecular data, we propose a new geographic and a new host record, for *Excipulariopsisnarsapurensis*.

Eurotiomycetes Eurotiomycetes O.E. Erikss & Winka, Myconet 1: 6 (1997)

Mycocaliciales Tibell & Wedin, Mycologia 92(3): 579 (2000)

*Mycocaliciaceae* A.F.W. Schmidt, Mitt. Staatsinst. Allg. Bot. Hamburg 13: 127 (1970)

*Chaenothecopsis* Vain., Acta Societatis pro Fauna et Flora Fennica 57 (1): 70 (1927)

Index Fungorum Registration Identifier: IF934

Type species: *Chaenothecopsis rubescens* Vain., Acta Societatis pro Fauna et Flora Fennica 57: 71 (1927)

**Figure 5:**
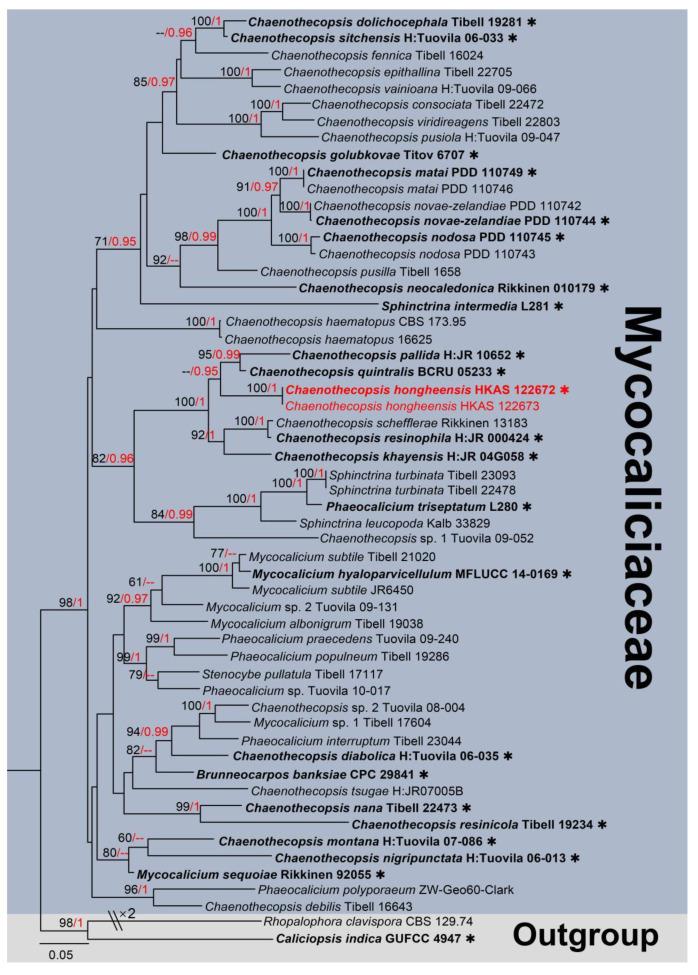
The phylogram based on a Maximum Likelihood analysis of combined LSU and ITS sequence datasets. Related sequences were taken from Tuovila et al. and Temu et al. [50,51,52]. The analyzed gene contains 56 fungal strains and 1443 total characters including gaps (LSU: 1–833 bp, ITS: 834–1443 bp). The tree topology of the ML resembles BI. The matrix had distinct alignment patterns, with the final ML optimization likelihood value of −14,762.676552 (ln). All free model parameters were estimated by the RAxML model, with 737 distinct alignment patterns and 23.22% of undetermined characters or gaps. Estimated base frequencies were as follows: A = 0.233526, C = 0.253106, G = 0.289045 and T = 0.224322, with substitution rates AC = 1.342770, AG = 2.445831, AT = 1.804595, CG = 0.988887, CT = 5.826613 and GT = 1.000000. The gamma distribution shape parameter alpha = 0.902547, and the Tree-Length = 3.752017. The final average standard deviation of split frequencies at the end of total MCMC generations was calculated as 0.009819 in BI analysis. The type strains are denoted in bold with the symbol “✱” at the ends, and newly introduced species in this study are denoted in red. The nodes provide bootstrap values of at least 60% (ML, left) and Bayesian posterior probabilities of at least 0.95 (BI, right); hyphens (-) signify values that are less than 60% in ML and less than 0.95 in BI. The blue and pale brown backgrounds were used to distinguish the selected species in Mycocaliciaceae and the outgroup.

Notes: *Chaenothecopsis* is a genus of the ascomycetes in the order Mycocaliciales [44], and to date, it accommodates a total of 101 records in Index Fungorum (2023) [45]. The majority of *Chaenothecopsis* taxa were previously known as resinicolous mycocalicioid taxa, which are saprotrophic on wood, resin (resinicolous), or associated with lichens as green algal symbionts [46,47]. In addition, *Chaenothecopsis polissica* as a fossil fungus was found in Rovno amber [48]. The taxa of this genus have raised, dark brown, branched or unbranched, straight to curved, apically fertile, broad apothecioid, synnematous fructifications. The sexual morph of *Chaenothecopsis* has oval to ellipsoidal, normally brown, ascospores with or without septa, and it is wrapped in slender cylindrical asci, while the asexual morph has rarely been reported before. Asexual morph fruiting bodies of *Chaenothecopsis* species are synnematous, with straight, parallel conidiophores compacted in the upper fertile parts, cylindrical, terminal or intercalary, tiny conidiogenous cells, and obovoid to clavate, aseptate, verruculose, catenated, olivaceous to brown conidia [49,50]. In this study, we provide the phylogenetic tree of *Chaenothecopsis* with their closely related groups (Figure 5).

**Figure 6:**
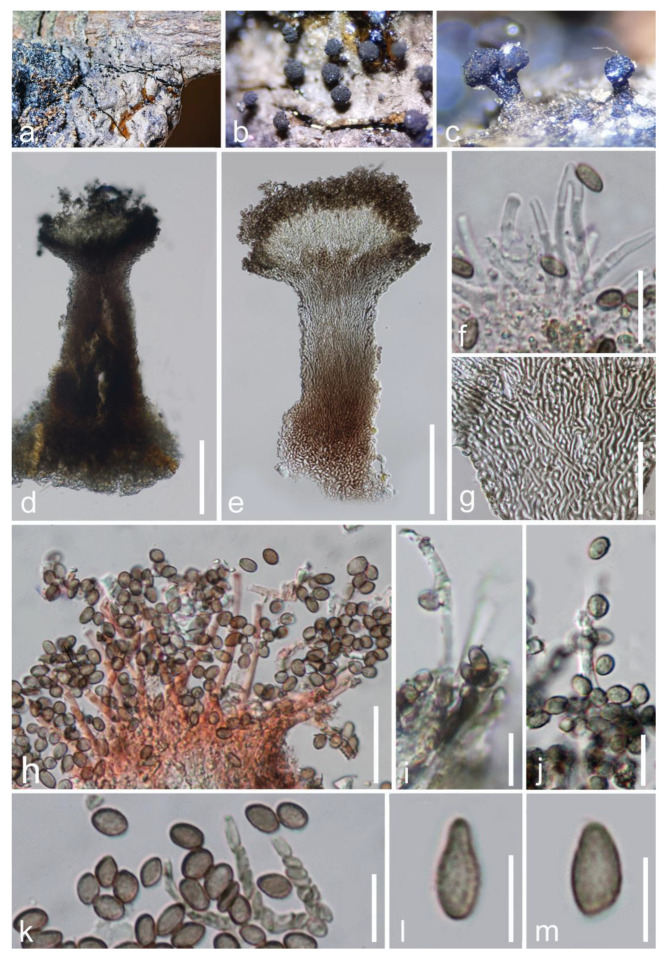
*Chaenothecopsis hongheensis* (HKAS 122672, holotype). (**a**–**c**) Synnemata on a dead branch of *Mangifera indica*; (**d**) vertical section of synnema; (**e**) synnema stained by Congo red reagent; (**f**,**h**–**j**) apical part of synnema; (**g**) mycelium of synnema; (**k**–**m**) conidia. Scale bars: (**d**,**e**) = 100 μm; (**f**,**g**) = 30 μm; (**h**) = 20um; (**i**–**k**) = 10 μm; (**l**,**m**) = 5 μm.

*Chaenothecopsis hongheensis* E.F. Yang & Tibpromma, sp. nov. (Figure 6).

Fungal Name number: FN571288

Etymology: The name reflects the location, Honghe, from where the holotype was collected.

Holotype: HKAS 122672

*Saprobic* on resin of dead branch of *Mangifera indica*. Sexual morph: Undetermined. Asexual morph: *Synnemata* erect on exudate or substrate, 380–470 μm ($\bar{x}$ = 420 μm, n = 10) high in total, 100–270 μm ($\bar{x}$ = 420 μm, n = 10) diam. at apical parts, dark grayish brown, straight or sometimes slightly flexuous, with obovoid to lentil-shaped head. *Stipe* 250–370 μm ($\bar{x}$ = 300 μm, n = 10) high, 60–120 μm ($\bar{x}$ = 90 μm, n = 10) wide, grayish black, relatively short, rarely branched, thickened at the bottom, composed of parallelly adpressed conidiophores. *Conidiophores* 45–65 μm ($\bar{x}$ = 55 μm, n = 20) high, 2–3 μm ($\bar{x}$ = 2.5 μm, n = 20) wide, compacted below, slightly flared on capitulum, numerous, sometimes apically branched, septate, flexuous, pale brown to brown with the maturity. *Conidiogenous cells* 1–2 μm wide, 2–3 μm long, lateral, polyblastic, terminal or intercalary, hyaline, short cannular to cylindrical, smooth. *Conidia* 4–5.5 × 2.5–4 μm ($\bar{x}$ = 4.5 × 3.5 μm, n = 20), obovoid to clavate, aseptate, flat to rounded at apex, verruculose, olivaceous to brown, rough walled, catenate, without a mucilaginous sheath.

Material examined: China, Yunnan Province, Honghe, Menglong Village, on the resin of a dead branch of *Mangifera indica* (102°50′11″ E, 23°41′01″ N, 500 m), 22 December 2020, E.F. Yang, erfu12 (Herb. HKAS 122673, holotype); isotype HKAS 122672. GenBank numbers: HKAS 122672 = ITS: OQ379009, LSU: OQ379420; HKAS 122673 = ITS: OQ379010, LSU: OQ379421.

Notes: In this study, we established a new resinicolous species of *Chaenothecopsis* growing on the resin of *Mangifera indica.* Our isolate fits very well within the common concept of *Chaenothecopsis,* with brown to dark brown, solitary, apothecioid, fructification emerging near to resin. However, only the asexual morph of our new isolate was available as synnematous with visible, erect, sometimes branched conidiophores on apical parts, conidiogenous cells lateral, terminal or intercalary, short granular, indistinct; and ovoid to obpyriform, olivaceous to brown, verruculose conidia. The asexual morph characteristics are distinguishable from other taxa in the *Chaenothecopsis* [53]. Additionally, the phylogenetic analyses also indicated that *Chaenothecopsis hongheensis* strains (HKAS 122672, 122673) are related to *C. pallida* (H: JR 10652) and *C. quintralis* (BCRU: 05233). A complete comparison of the 789 nucleotides of the LSU gene region from *C. pallida* (H: JR 10652) with our isolates revealed 26 base pair differences (3.29%) with 0 gaps. A detailed comparison of the 514 nucleotides of the ITS gene region from our isolate and *C. pallida* (H: JR 10652) revealed 68 base pair differences with 29 gaps. As our isolate is well separated from *Chaenothecopsis pallida* (H: JR 10652) and *C. quintralis* (BCRU: 05233) in morphology and phylogeny, we introduce *C. hongheensis* as a distinct new species with a unique anamorph.

Sordariomycetes O.E. Erikss & Winka 1997

Sordariales Chadef. ex D. Hawksw & O.E. Erikss., Syst. Ascom. 5(1): 182 (1986)

Helminthosphaeriaceae Samuels, Cand &Magni 1997

*Hilberina* Huhndorf & A.N. Mill., Mycological Research 108 (1): 31 (2004)

Index Fungorum Registration Identifier: IF28830

Type species: *Hilberina caudata* (Fuckel) Huhndorf & A.N. Mill., Mycological Research 108 (1): 31 (2004)

**Figure 7:**
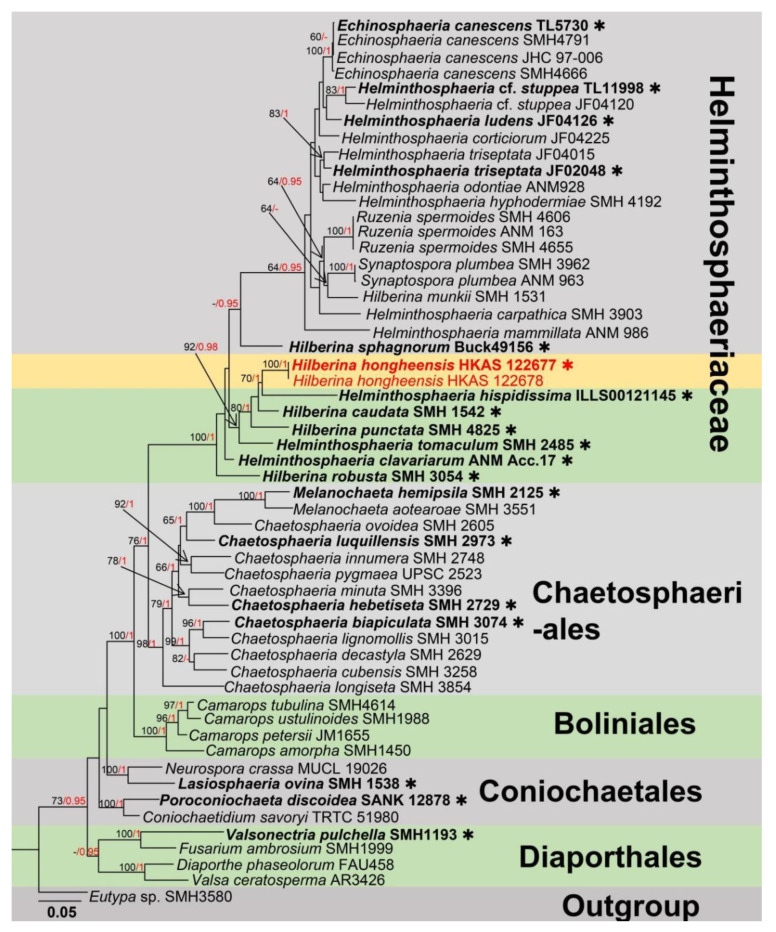
The phylogram based on a Maximum Likelihood analysis of combined LSU and *tub2* sequence datasets. Related sequences were taken from Hernández-Restrepo et al. [57]. The analyzed gene contains 54 fungal strains and 1688 total characters including gaps (LSU: 1–849 bp, *tub2*: 850–1688 bp). The tree topology of the ML resembles BI. The matrix had distinct alignment patterns, with the final ML optimization likelihood value of −15,023.931027 (ln). All free model parameters were estimated by the RAxML model, with 705 distinct alignment patterns and 10.51% of undetermined characters or gaps. Estimated base frequencies were as follows: A = 0.211401, C = 0.291809, G = 0.301153 and T = 0.195636, with substitution rates AC = 0.570232, AG = 2.419550, AT = 1.285900, CG = 1.382428, CT = 6.918864 and GT = 1.000000. The gamma distribution shape parameter alpha = 0.633379, and the Tree-Length = 2.181051. The final average standard deviation of split frequencies at the end of total MCMC generations was calculated as 0.009875 in BI analysis. The type strains are denoted in bold with the symbol “✱” at the ends, and newly introduced species in this study are denoted in red. The nodes provide bootstrap values of at least 60% (ML, left) and Bayesian posterior probabilities of at least 0.95 (BI, right); hyphens (-) signify values that are less than 60% in ML and less than 0.95 in BI. The greenish and pale brown backgrounds were used to distinguish different groups, while the yellow background indicates new isolates obtained in this study.

Notes: The genus *Hilberina* was re-described by Miller and Huhndorf [54,55], with *H. caudata* as the type species. The sexual morphological characteristics of this genus are as follows: Often associated with dead woody materials, presenting superficial, subglobose to ovoid ascomata with thick walled, brown, long setae and papillate ostiole; oblong, cylindrical asci contain subglobose, septate or aseptate, hyaline ascospores without gelatinous appendages. Molecular data are available for only five species (*Hilberina caudata*, *H. sphagnorum*, *H. punctata*, *H. robusta* and *H. munkii*). The members of *Hilberina* are very closely related to *Synaptospora, Ruzenia* and *Helminthosphaeria* (Helminthosphaeriaceae) in morphological characteristics and phylogenetic placements [56,57]. To date, 20 species records of *Hilberina* are in Species Fungorum (2023) [58], and many of them were transferred from *Lasiosphaeria* and *Sphaeria* based on morphology [56]. One species of *Hilberina* (*H. breviseta*) associated with submerged wood in Dianchi Lake has been reported from Yunnan Province, China [59]. In this study, we updated the multi-gene phylogenetic tree of *Hilberina* as shown in Figure 7.

**Figure 8:**
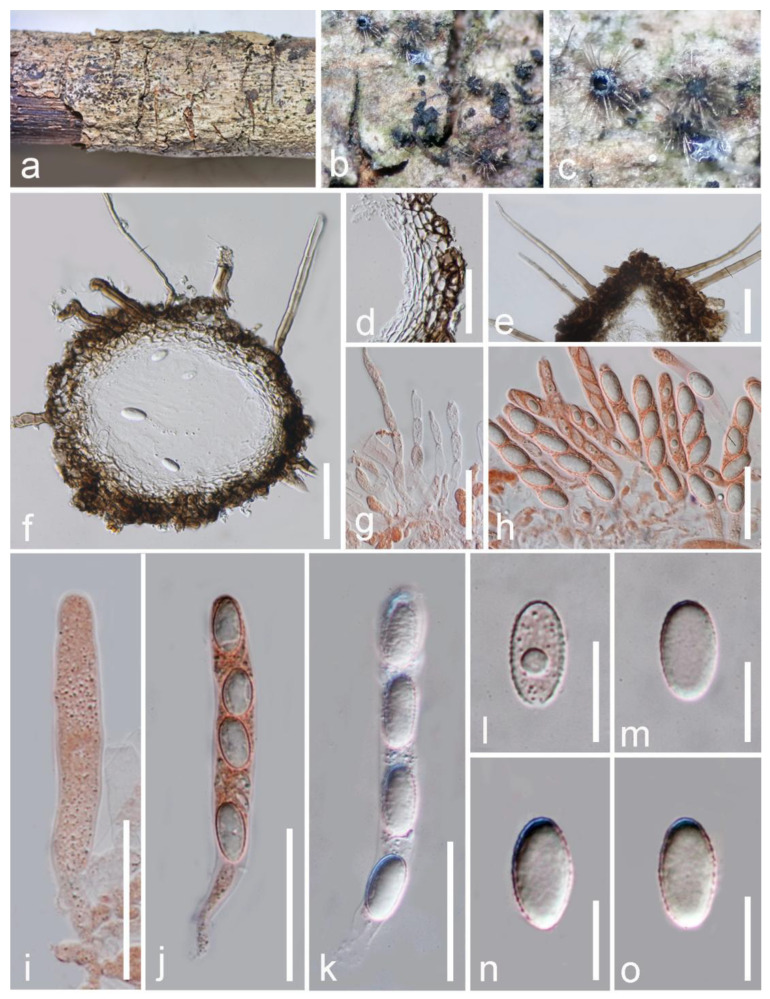
*Hilberina hongheensis* (HKAS 122677, holotype). (**a**) Ascomata attached on host surface; (**b**,**c**) ascomata magnified under a stereo microscope; (**d**,**f**) vertical section of ascomata wall; (**e**) apical section of ascoma with setae; (**g**) paraphyses; (**h**–**j**) asci stained by Congo red reagent; (**k**) asci; (**l**–**o**) ascospores. Scale bars: (**d**,**f**) = 50 μm; (**e**,**g**,**k**) = 25 μm; (**h**–**j**) = 20 μm, (**l**–**o**) = 10 μm.

*Hilberina hongheensis* E.F. Yang & Tibpromma, sp. nov. (Figure 8)

Fungal Name number: FN571289

Etymology: The name reflects the location, Honghe, from where the holotype was collected.

Holotype: HKAS 122677

*Saprobic* on a dead branch of *Mangifera indica.* Sexual morph: *Ascomata* 135–160 × 145–180 μm ($\bar{x}$ = 150 × 160 μm, n = 10), superficial, subglobose, scattered, solitary, carbonaceous black, setiferous on the surface of the substrate, with long black papilla visible at the surface, with a poorly developed ostiole. *Setae* 7–10 high, pale brown to brown, sub-hyaline at the tip, septate, straight to slightly flexuous, smooth, with a narrow acute apex. *Ascomata wall* 25–35 ($\bar{x}$ = 31 μm, n = 10) μm wide, dark brown, multilayered, comprising hyaline cells of *textura angularis* inwardly, with outer layers appearing dark brown and thick walled. *Hamathecium* composed of 2–5 μm-wide, septate, cylindrical, filamentous, unbranched paraphyses constricted at septa. *Asci* 70–85 × 7–10 μm ($\bar{x}$ = 75 × 9 μm, n = 20), eight spored, unitunicate, often four spored when immature, well-developed when mature, cylindrical, oblong, hyaline, slightly shining, short pedicellate, apically rounded, thick walled; sometimes with four poorly developed ascospores in each ascus. *Ascospores* 13–16 × 6–9 μm ($\bar{x}$ = 15 × 8 μm, n = 30), uniseriately overlapping, oval, hyaline, thin and rough walled, verruculose, with oil droplets disappearing on maturity, without a gelatinous sheath and appendages. Asexual morph: Undetermined.

Material examined: China, Yunnan Province, Baoshan City, Longling County, on a dead and decaying branch of *Mangifera indica* (99°16′80″ E, 25°12′23″ N, Elevation: 800 m) 27 December 2019, E.F. Yang, MB004 (HKAS 122677, holotype); isotype HKAS 122678, GenBank numbers: HKAS 122677 = LSU: OQ379422, *tub2*: OQ379003; HKAS 122678 = LSU: OQ379423, *tub2*: OQ379004, *tef1*-*α*: OQ379002.

Notes: Based on morphology, our isolate fits well within the generic concepts of *Hilberina* rather than *Helminthosphaeria* and closely resembles the type species of *Hilberina*, (*H. caudata*). However, *H. caudata* was originally described as having ascomata with setae, papillate neck; cylindrical, eight-spored asci with a refractive, inamyloid apical ring; septate, cylindrical, geniculate, hyaline to pale brown ascospores with one end tapering to a thin tip [55,60]. Our new isolate differs from *H. caudata* by subglobose to broadly fusiform, one-celled ascospores. The BLASTn search results and percent sequence of LSU and *tub2* show similarity to *H. caudata* strains SMH 1542 with 95.8% and 98.7%, respectively. Consequently, our isolates are identified as a distinct novel species in *Hilberina* based on morphological and phylogenetic studies.

Sordariomycetes O.E. Erikss & Winka 1997

Delonicicolales R.H. Perera, Maharachch & K.D. Hyde 2017

Delonicicolaceae R.H. Perera, Maharachch & K.D. Hyde, in Perera, Maharachchikumbura, Jones, Bahkali, Elgorban, Liu, Liu & Hyde, Cryptog. Mycol. 38(3): 334 (2017)

*Delonicicola* R.H. Perera, Maharachch & K.D. Hyde, Cryptogamie, Mycologie 38: 334 (2017).

Index Fungorum Registration Identifier: IF553771

Type species: *Delonicicola siamense* R.H. Perera, Maharachch. & K.D. Hyde, Cryptogamie, Mycologie 38: 335 (2017)

**Figure 9:**
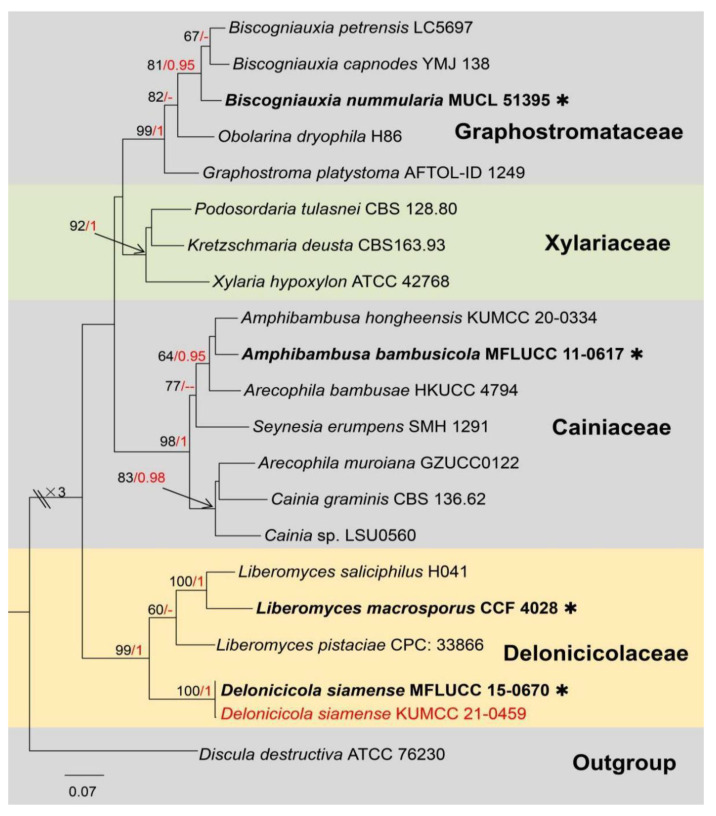
The phylogram based on a Maximum Likelihood analysis of combined LSU, ITS and *rpb2* sequence datasets. Related sequences were taken from Perera et al. [61]. The analyzed gene contains 21 fungal strains and 2500 total characters including gaps (LSU: 1–857 bp, ITS: 858–1463 bp, *rpb2*: 1464–2500 bp). The tree topology of the ML resembles BI. The matrix had distinct alignment patterns, with the final ML optimization likelihood value of −12,817.082414 (ln). All free model parameters were estimated by the RAxML model, with 987 distinct alignment patterns and 40.49% of undetermined characters or gaps. Estimated base frequencies were as follows: A = 0.258092, C = 0.230665, G = 0.269431 and T = 0.241812, with substitution rates AC = 1.743230, AG = 3.960524, AT = 1.741408, CG = 1.170575, CT = 7.682505 and GT = 1.000000. The gamma distribution shape parameter alpha = 0.453818, and the Tree-Length = 2.619478. The final average standard deviation of split frequencies at the end of total MCMC generations was calculated as 0.009681 in BI analysis. The type strains are denoted in bold with the symbol “✱” at the ends, and newly introduced species in this study are denoted in red. The nodes provide bootstrap values of at least 60% (ML, left) and Bayesian posterior probabilities of at least 0.95 (BI, right); hyphens (-) signify values that are less than 60% in ML and less than 0.95 in BI. The bluish and pale brown backgrounds were used to distinguish different family groups, while the yellow background indicates the Delonicicolaceae and new isolate.

Notes: The genus *Delonicicola* was first established for a sexual morph, with *D. siamense* as the type species. The type species was found as saprobic on dried seed pods of *Delonix regia* in Thailand, and full descriptions and morphological characteristics were offered by Perera et al. [61]. Currently, *Delonicicola* is placed under the family *Delonicicolaceae* along with *Liberomyces* and *Furfurella* [62,63]. The sexual morph of *Delonicicola* was introduced as having grouped, black, conspicuous, raised pseudo-stromata, with multi-loculate, aggregated, immersed, globose to conical, ostiolate, ascomata; paraphyses filamentous, aseptate, unbranched; eight-spored, unitunicate, clavate, short pedicellate asci; ascospores are one or two seriate, one septate, blunt at ends, straight, hyaline, and smooth walled [61]. The asexual morph of *Delonicicola* has so far not been reported and herein introduced for the first time. *Conidiomata* immersed, multi-loculate, with a prolonged neck; *Conidiophores* aseptate, cylindrical, straight to slightly curved, sometimes branched. *Conidiogenous cells* holoblastic, with a cylindrical lumen. *Conidia* lunate, zero-to-one septate, hyaline, curved, and tapering towards apex. The phylogenic relationships of *Delonicicola* (Delonicicolaceae) and closely related genera/families are shown in Figure 9.

**Figure 10:**
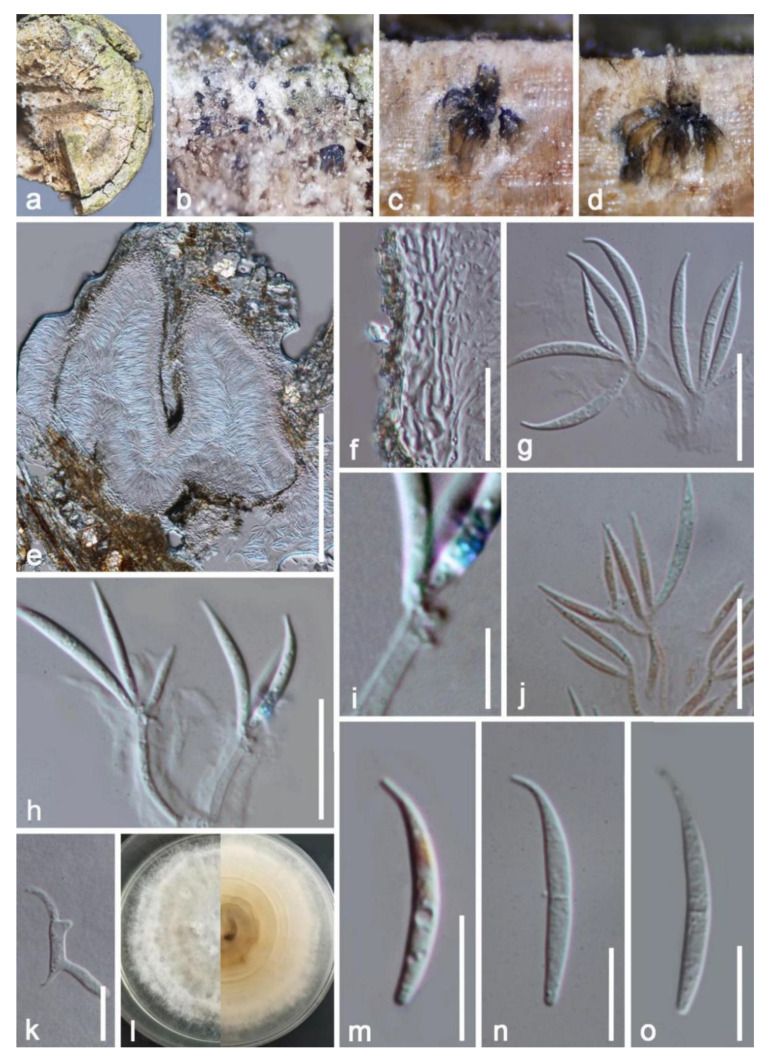
*Delonicicola siamense* (HKAS 122662). (**a**,**b**) The appearance of the natural substrate; (**c**–**e**) cross section of conidiomata; (**f**) conidiomata wall; (**g**,**h**) conidia with conidiophore; (**j**) conidiophore and conidia stained by Congo red reagent; (**i**) close-up of conidiogenous cells; (**k**) germinated conidium; (**l**) colonies on PDA; (**m**–**o**) Conidia. Scale bars: (**e**) = 200 μm; (**f**,**g**) = 20 μm; (**j**,**h**) = 15 μm; (**m**–**o**) = 10 μm; (**i**) = 5 μm.

*Delonicicola siamense* R.H. Perera, Maharachch & K.D. Hyde, Cryptogamie, Mycologie 38: 335 (2017) (Figure 10)

Index Fungorum Registration Identifier: IF553771

*Saprobic* on dead stem of *Mangifera indica*. Sexual morph: Available in Perera et al. [61]. Asexual morph: *Conidiomata* 190–340 × 120–170 μm ($\bar{x}$ = 265 × 150 μm, n = 20), pycnidial, fully immersed in plant tissues, with apical part of prolonged neck visible above the surface, multi-loculate, conspicuous at surface, dark brown. *Conidiomata wall* 11–17 μm ($\bar{x}$ = 14 μm, n = 20), comprising thick-walled, brown cells of *textura epidermoidea,* 3.5–6.5 μm ($\bar{x}$ = 5 μm, n = 20) wide at outer layers, merged with the host. *Paraphyses* absent. *Conidiophores* 15–30 μm ($\bar{x}$ = 18 μm, n = 20) high, 1–2 μm ($\bar{x}$ = 1.5 μm, n = 20) wide, hyaline, branched, septate, cylindrical, straight to slightly flexuous. *Conidiogenous cells* 1–2 μm high, 0.5–1 μm wide, nodose at the tip, polyblastic, sympodial, slightly bulged at the apex. *Conidia* 19–26 × 1.5–3 μm ($\bar{x}$ = 22 × 2.5 μm, n = 20), lunate, zero-to-one septate, with a central septum, granulate, smooth, truncate at base, curved above half and tapering to apex.

Culture characters: Conidia formed germ tube on PDA within 24 h at room temperature, fast-growing and colonies reaching around 30 mm within half a month, colonies circular, flat to effuse, slightly radiating, gray to whitish outwardly, with entire margin; reverse: reddish brown at the center, becoming white towards margin. Without pigments released in PDA.

Substratum: Seed pods of *Delonix regia* [61]; a dead stem of mango (*Mangifera indica*) (this study).

Distribution: Chiang Rai, Thailand [61]; Yunnan, China (this study).

Material examined: China, Yunnan Province, Honghe, Menglong Village, on a dead stem of *Mangifera indica* (102°50′11″ E, 23°41′01″ N, 500 m), 22 December 2020, E.F. Yang, MB012-1 (Herb. HKAS 122662); living culture KUMCC 21-0459. GenBank numbers: KUMCC 21-0459 = ITS: OQ379013, LSU: OQ379424, *rpb2*: OQ378992.

Notes: The genus *Delonicicola*, with *D. siamense* as the type species, was introduced by Perera et al. [61], and it is so far known only from its sexual morph. Multigene phylogenetic analysis (ML and BI) showed our isolate (KUMCC 21-0459) clusters with *D. siamense* (MFLUCC 15-0670) with high statistical support (100% in ML, 1 in BI; Figure 9). Moreover, the BLASTn results of ITS, LSU and *rpb2* indicated that our isolate belongs to *Delonicicola siamense* with a similarity of >99%. Morphologically, the conidia of *D. siamense* (KUMCC 21-0459) are zero-to-one septate at the central, hyaline, granulate and slightly curved. Our isolate (KUMCC 21-0459) is identified as *Delonicicola siamense* based on morphological examinations together with phylogenetic analyses. In addition, this is the first report of an asexual morph of *D. siamense* and a new host and country record.

Sordariomycetes O.E. Erikss & Winka 1997

Coronophorales Nannf., Nova Acta R. Soc. Scient. upsal., Ser. 4 8(no. 2): 54 (1932)

Nitschkiaceae Nannf., Nova Acta R. Soc. Scient. upsal., Ser. 4 8(no. 2): 56 (1932)

*Fracchiaea* Sacc., Nuovo Giornale Botanico Italiano 5: 285 (1873)

Index Fungorum Registration Identifier: IF2008

Type species: *Fracchiaea heterogenea* Sacc., Nuovo Giornale Botanico Italiano 5: 285 (1873)

**Figure 11:**
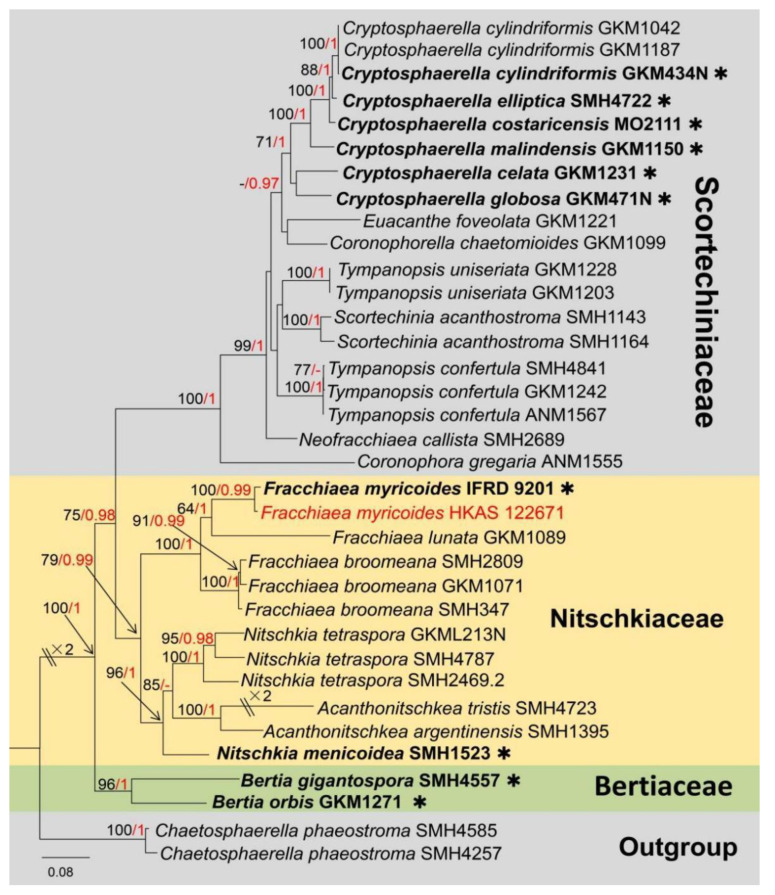
The phylogram based on a Maximum Likelihood analysis of combined LSU, *tef1*-*α* and *rpb2* sequence datasets. Related sequences were taken from Huang et al. [66]. The analyzed gene contains 35 fungal strains and 2873 total characters including gaps (LSU: 1–964 bp, *tef1*-*α*: 965–1758 bp, *rpb2*: 1759–2873 bp). The tree topology of the ML resembles BI. The matrix had distinct alignment patterns, with the final ML optimization likelihood value of −22,781.891350 (ln). All free model parameters were estimated by the RAxML model, with 1017 distinct alignment patterns and 15.66% of undetermined characters or gaps. Estimated base frequencies were as follows: A = 0.236282, C = 0.284832, G = 0.287302 and T = 0.191584, with substitution rates AC = 1.282471, AG = 3.774835, AT = 1.599302, CG = 1.361280, CT = 8.941815 and GT = 1.000000. The gamma distribution shape parameter alpha = 0.719878, and the Tree-Length = 3.502849. The final average standard deviation of split frequencies at the end of total MCMC generations was calculated as 0.009364 in BI analysis. The type strains are denoted in bold with the symbol “✱” at the ends, and newly introduced species in this study are denoted in red. The nodes provide bootstrap values of at least 60% (ML, left) and Bayesian posterior probabilities of at least 0.95 (BI, right); hyphens (-) signify values that are less than 60% in ML and less than 0.95 in BI. The greenish and pale brown backgrounds were used to distinguish different family groups, while the yellow background indicates the family Nitschkiaceae and new isolate.

Notes: *Fracchiaea*, as typified by *F. heterogenea,* is nested in the Nitschkiaceae [64], and the genus is basal to the clade of *Acanthonitschkea* and *Nitschkia* in the phylogeny (Figure 11). This genus includes 35 species, but only three species (*Fracchiaea broomeana, F. lunata,* and *F. myricoides*) have molecular data [65,66]. The species of *Fracchiaea* are commonly saprobic on wood. The sexual morph of *Fracchiaea* differs by being immersed to erumpent, carbonaceous to coriaceous, black, turbinate ascomata; polysporous, unitunicate, clavate to cylindrical, pedicellate or sessile asci; hyaline to yellowish, ellipsoidal to cylindrical to allantoid, slightly flexuous, numerous, zero-to-one septate ascospores. However, asexual morph is undetermined [65,66].

**Figure 12:**
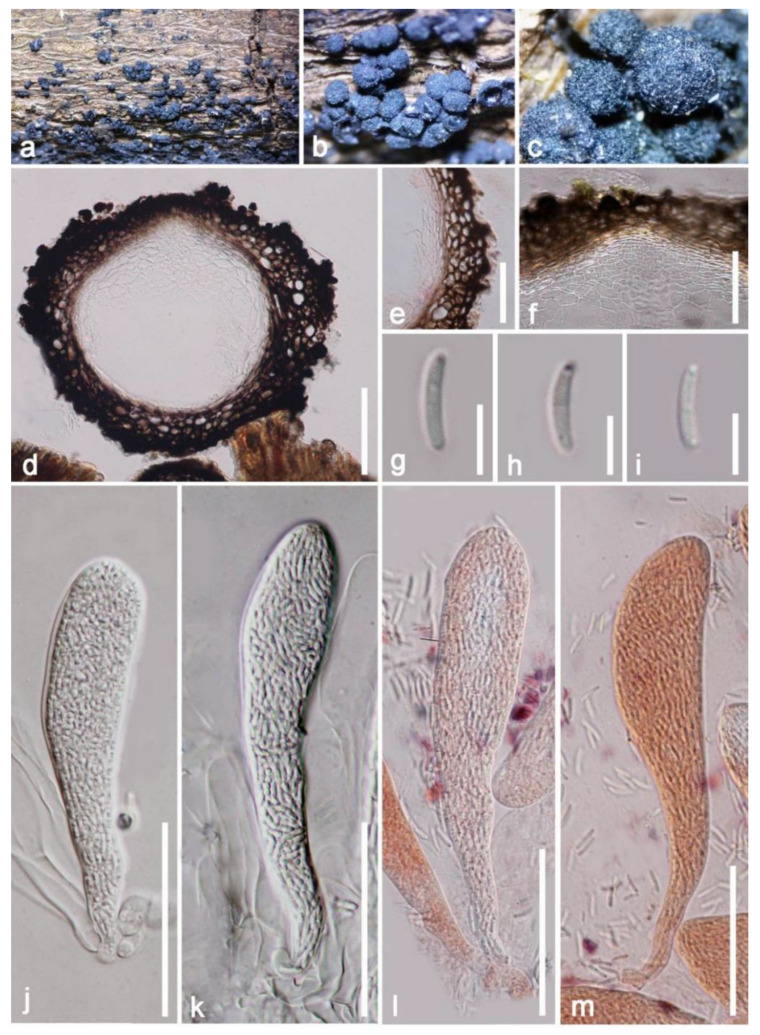
*Fracchiaea myricoides* (HKAS 122671). (**a**) Numerous ascomata on a bark of mango; (**b**,**c**) close-up of ascomata; (**d**) vertical section of ascomata; (**e**) ascomata wall; (**f**) ascomata wall at the apex; (**j**–**m**) asci with or without stained by Congo red reagent; (**g**–**i**) ascospore. Scale bars: (**d**) =100 μm; (**e**,**f**,**j**–**m**) = 50 μm; (**g**–**h**) = 5 μm.

*Fracchiaea myricoides* (H.X. Wu & K.D. Hyde) S.K. Huang & K.D. Hyde, Mycosphere 12 (1): 920 (2021) (Figure 12)

Index Fungorum Registration Identifier: IF558200

*Saprobic* on a dead bark of *Mangifera indica*. Sexual morph: *Ascomata* up to 280–330 × 310–420 μm ($\bar{x}$ = 275 × 365 μm, n = 10), perithecial, clustered in small to large groups, superficial or erumpent, globose to subglobose, with poorly developed ostiole at the center, rough at the surface, tuberculate, dark brown to carbonaceous black. *Ascomata wall* 45–70 μm wide, multilayered, thick walled, composed of multilayered dark brown-walled cells of *textura globosa*; rough at the outer surface, comprised of hyaline pseudoparenchymatic cells of *textura angularis* in inner layers 15–55 μm ($\bar{x}$ = 35 μm, n = 20). *Paraphyses* absent. *Asci* 100–150 × 12–26 μm ($\bar{x}$ = 125 × 19 μm, n = 20), numerous, unitunicate, polysporous, oblong, cylindric clavate, short pedicellate, round or blunt at apex, without a visible discharge mechanism. *Ascospores* 6–8 × 1.5–2 μm ($\bar{x}$ = 7 × 2 μm), numerous, crowded, hyaline, cylindrical to allantoid, slightly curved, aseptate, smooth walled, with granules. Asexual morph: Undetermined.

Substratum: Dead wood of an unidentified plant [67] dead mango bark (this study).

Distribution: Yunnan Province, China ([67], this study).

Material examined: China, Yunnan Province, Honghe, Menglong Village, on a dead bark of *Mangifera indica* (102°50′11″ E, 23°41′01″ N, 500 m), 22 December 2020, E.F.Yang, HHE020 (Herb. HKAS 122671). GenBank numbers: HKAS 122671 = ITS: OQ379014, LSU: OQ379425, *tef1*-*α*: OQ378999, *rpb2*: OQ378993.

Notes: Our isolate fits well within the concept of *Fracchiaea* by forming gregarious, carbonaceous to coriaceous, black ascomata without ostiole on a woody surface; ascomata wall comprising *textura angularis* to *textura prismatica* cells; without paraphyses; zero-to-one septate, numerous ascospores wrapped in a clavate-to-cylindrical ascus. Based on multi-gene phylogenetic analysis of combined LSU, *tef1*-*α* and *rpb2* sequence data, our isolate (HKAS 122671) is well clustered with the ex-type strain of *F. myricoides* (IFRD 9201) with high and reliable statistical support (Figure 11). In addition, our isolate and *F. myricoides* (IFRD 9201) have similarly sized ascomata, asci and ascospores (ascomata: $\bar{x}$ = 275 × 365 μm vs. $\bar{x}$ = 383 × 308 μm; asci: $\bar{x}$ = 125 × 19 μm vs. $\bar{x}$ = 119 × 22 μm; ascospore: $\bar{x}$ = 7 × 2 μm vs. $\bar{x}$ = 7.9 × 1.7 μm) [66,67]. In addition, in mega BLASTn search using the LSU sequence, the closest matches of *F. myricoides* (IFRD 9201) in NCBI’s GenBank nucleotide database showed 99.06% similarity (Identities = 951/960 bp; Gaps = 0). Unfortunately, the *tef1*-*α* and *rpb2* molecular data of *Fracchiaea myricoides* (IFRD 9201) were unavailable. Nevertheless, we regard our isolate as *F. myricoides*, and this is the first time to report *F. myricoides* from *Mangifera indica* based on morphological examination and phylogenetic analysis. In addition, *Coronophora myricoides* originally collected from unidentified woody debris in China has been transferred to the *Fracchiaea* based on phylogenetic analysis and morphological comparison [66].

Dothideomycetes sensu O.E. Erikss & Winka 1997

Monoblastiales Lücking, M.P. Nelsen & K.D. Hyde, in Hyde et al., Fungal Diversity 63: 8 (2013)

Monoblastiaceae Walt. Watson, New Phytol. 28: 106 (1929)

*Heleiosa* Kohlm., Volkm.-Kohlm & O.E. Erikss., Can. J. Bot. 74: 1830 (1996)

Index Fungorum Registration Identifier: IF27767

Type species: *Heleiosa barbatula* Kohlm., Volkm.-Kohlm & O.E. Erikss. 1996.

**Figure 13:**
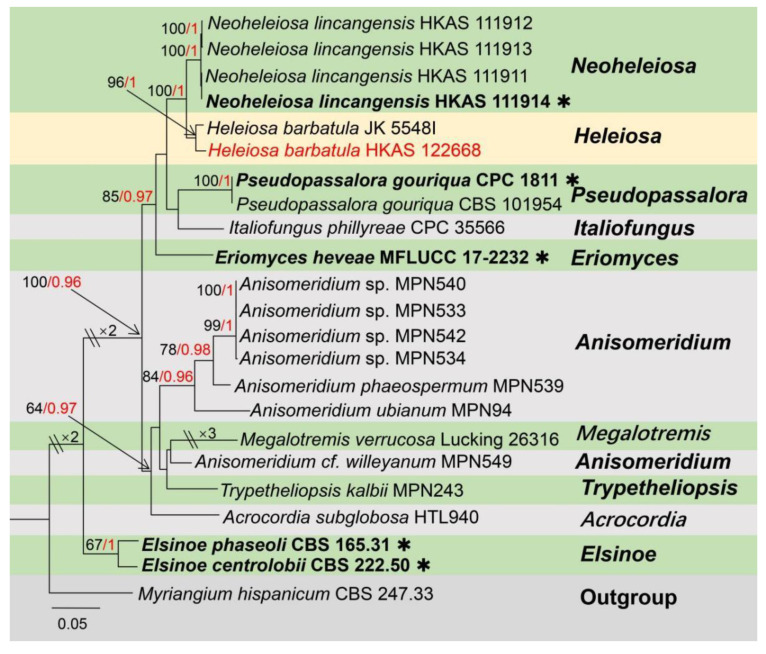
The phylogram based on a Maximum Likelihood analysis of combined LSU, SSU, ITS and *tef1-α* sequence datasets. Related sequences were taken from Mortimer et al. [71]. The analyzed gene contains 23 fungal strains and 3280 total characters including gaps (LSU: 1–860 bp, SSU: 861–1903 bp, ITS: 1904–2388 bp, *tef1-α*: 2389–3280 bp). The tree topology of the ML resembles BI. The matrix had distinct alignment patterns, with the final ML optimization likelihood value of −11,066.133147 (ln). All free model parameters were estimated by the RAxML model, with 822 distinct alignment patterns and 43.17% of undetermined characters or gaps. Estimated base frequencies were as follows: A = 0.234980, C = 0.257617, G = 0.290520 and T = 0.216883, with substitution rates AC = 1.135176, AG = 1.801869, AT = 1.203774, CG = 1.318707, CT = 7.467159 and GT = 1.000000. The gamma distribution shape parameter alpha = 0.211437, and the Tree-Length = 1.143995. The final average standard deviation of split frequencies at the end of total MCMC generations was calculated as 0.009737 in BI analysis. The type strains are denoted in bold with the symbol “✱” at the ends, and newly introduced species in this study are denoted in red. The nodes provide bootstrap values of at least 60% (ML, left) and Bayesian posterior probabilities of at least 0.95 (BI, right); hyphens (-) signify values that are less than 60% in ML and less than 0.95 in BI. The greenish and pale brown backgrounds were used to distinguish different genera groups, while the yellow background indicates the genus *Heleiosa*.

Notes: The monotypic genus *Heleiosa* was established by Kohlmeyer in 1996 [68], with *H. barbatula* as the type species. *Heleiosa barbatula* was collected from *Junciroemeriani* in the USA (North Carolina). The morphological characteristics of this genus differ by immersed, papillate, clypeate ascomata, without neck, sparse periphyses around ostiolar canal; ascomata wall is light colored, thin, comprising *textura angularis* cells; pseudoparaphyses branched, anastomosis, extending over the asci; asci are four-to-eight spored; producing fusiform, brown, one-celled ascospores, with numerous cilia-like sub-apical appendages at each end [68]. Lately, the phylogenetic analysis also supported *Heleiosa* as a separate genus in the Monoblastiaceae. It has been speculated that *H. barbatula* and *Funbolia dimorpha*, as non-lichen-forming taxa, strongly support a close relationship with the Monoblastiaceae and that they could provide insight into the evolution of the lichen-forming habit [69,70]. Moreover, *Neoheleiosa* was introduced close to *Heleiosa* (type strain: JK5548I) with high statistical support in phylogenetic trees [71]. *Heleiosa* is still a poorly studied group, and only one single species with a sexual morph is available. The phylogeny of *Heleiosa* and relative genera is shown in Figure 13.

**Figure 14:**
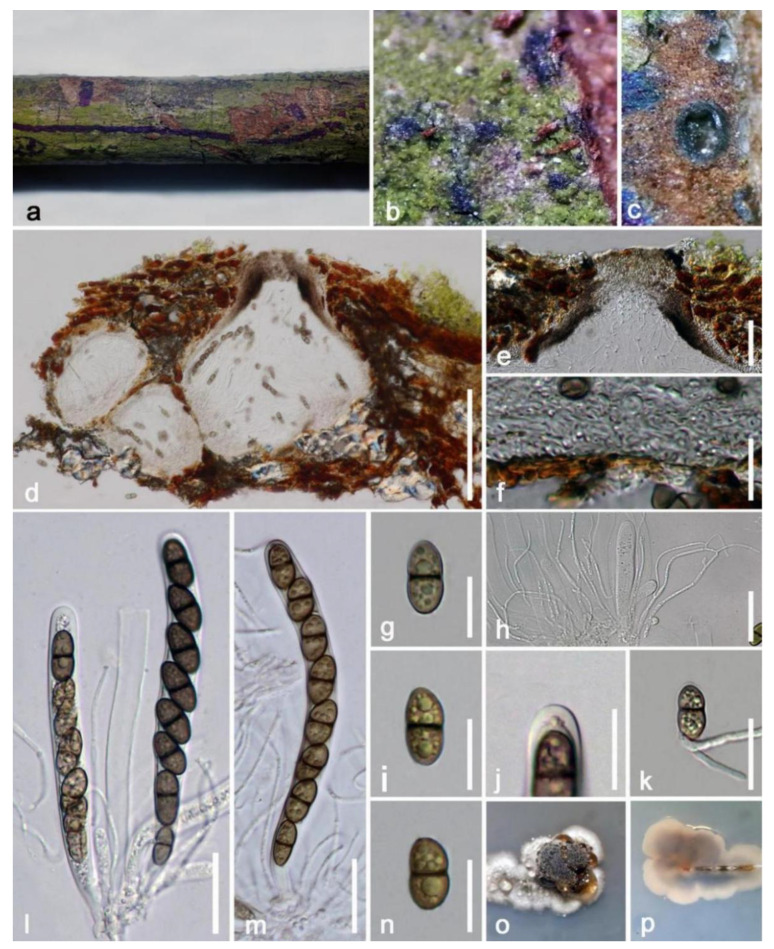
*Heleiosa barbatula* (HKAS 122668). (**a**) Dead branch of mango; (**b**) close-up of the immersed ascomata; (**c**) transverse section of ascomata; (**d**) vertical section of ascomata; (**e**) section of the ostiole; (**f**) ascomata wall; (**h**) pseudoparaphyses; (**l**,**m**) asci (note bitunicate nature); (**g**,**i**,**n**) ascospores; (**j**) apical chamber of ascus; (**k**) germinated ascospore; (**o**,**p**) colony on PDA. Scale bars: (**d**) = 150 µm; (**e**) = 50 µm; (**h**) = 30 µm; (**l**,**m**) = 25 µm; (**k**) = 20 µm; (**f**,**g**,**i**,**n**,**j**) = 10 µm.

*Heleiosabarbatula* Kohlm., Volkm.-Kohlm & O.E. Erikss., Canadian Journal of Botany 74: 1830 (1996) (Figure 14)

Index Fungorum Registration Identifier: IF436638

*Saprobic* on a dead branch of *Mangifera indica.* Sexual morph: *Ascomata* (excluding ostiole) 180–280 × 200–280 μm ($\bar{x}$ = 230 × 240 μm, n = 20), globose to ampulliform, brown to dark brown, aggregated, sometimes solitary, uni- or multiloculate, fully immersed beneath host epidermis when immature, raised with erumpent neck when mature, visible as black scars on the surface. *Ostiole* 45–60 × 100–125 μm ($\bar{x}$ = 50 × 115 µm, n = 20), brown, central, cylindrical, flat at the apex. *Perdium* 25–40 μm ($\bar{x}$ = 34 µm, n = 20) wide, multilayered, hyaline to lightly brown comprising hyaline cells of *textura prismatica* in inner layers, thick walled, hyaline to lightly brown, outermost cells merged with plant tissues. *Hamathecium* generated from a gelatinous matrix, composed of 1–2 µm-wide, filamentous, hyaline, numerous, branched, septate, pseudo-paraphyses. *Asci* 100–130 × 8–13 µm ($\bar{x}$ = 120 × 11 µm, n = 30), eight spored, bitunicate, short pedicellate, cylindrical, with a furcated base, apically rounded with an indistinct ocular chamber. *Ascospores* 14–18 × 6–9 µm ($\bar{x}$ = 15 × 8 µm, n = 30), uniseriate, one septate, ellipsoidal, slightly constricted at the septum, hyaline to green-brown, with oil droplets, obtuse ends, smaller upper cell, thick walled.

Culture characteristics: On PDA media, ascospores randomly produced germ tubes near obtuse ends within 20 h, taking two months to develop 10 mm-diam. Colonies at room temperature slowly; colonies circular, fluffy, convex, brown, dense; reverse: dark reddish brown, white at the margin, without pigments produced in PDA.

Substratum: Rush of *Junciroemeriani* [68]; dead branch of *Mangifera indica* (this study).

Distribution: North Carolina, Virginia, the USA [68]; Yunnan Province, China (this study).

Material examined: China, Yunnan Province, Baoshan City, Longling County, on a dead branch of *Mangifera indica* (99°16′80″ E, 25°12′23″ N, Elevation: 800 m) 27 December 2019, E.F. Yang, MB012 (HKAS 122668), living culture, KUMCC 21-0462. GenBank number: HKAS 122668 = ITS: OQ379015, LSU: OQ379426, SSU: OQ372923, *tef1-α*: OQ379000.

Notes: *Heleiosa* has only one species (*H. barbatula*) as described by Kohlmeyer et al. [68]. Our isolate fits well within the concept of *Heleiosa barbatula* by producing immersed, subglobose ascomata; asci are oblong, with an ocular chamber at the apex, and have a similar size (75–100 × 9–11 µm vs. 100–130 × 8–13 µm); ascospores are ellipsoidal to cylindrical, one septate, with obtuse ends, green brown to brown, with oil globules, and have a similar size (16.5–22.5 (–24) × 5.5–7 µm vs. 14–18 × 6–9 µm). In addition, a comparison of the LSU region of our isolate (KUMCC 21-0462) and *Heleiosa barbatula* (JK 5548I) reveals 98% similarity, and the SSU region reveals 100% similarity. Although *Heleiosa barbatula* (JK 5548I) lacks ITS and *tef1*-*α* sequencing data, the morphological characteristics and multi-gene phylogenetic results fully support our isolate as the same species as *H. barbatula* (Figure 13). Therefore, we report *Heleiosa barbatula* (KUMCC 21-0462) on *Mangifera indica* as a new host and country record.

Dothideomycetes sensu O.E. Erikss & Winka 1997

Pleosporales Luttr. ex M.E. Barr, Prodr. Cl. Loculoasc. (Amherst): 67 (1987)

Hermatomycetaceae Locq., Mycol. gén. struct. (Paris): 202 (1984)

*Hermatomyces* Speg., Anales del Museo Nacional de Historia Natural Buenos Aires ser. 3, 13: 445 (1911)

Index Fungorum Registration Identifier: IF8517

Type species: *Hermatomyces tucumanensis* Speg., Anales del Museo Nacional de Historia Natural Buenos Aires ser. 3, 13: 446 (1911)

**Figure 15:**
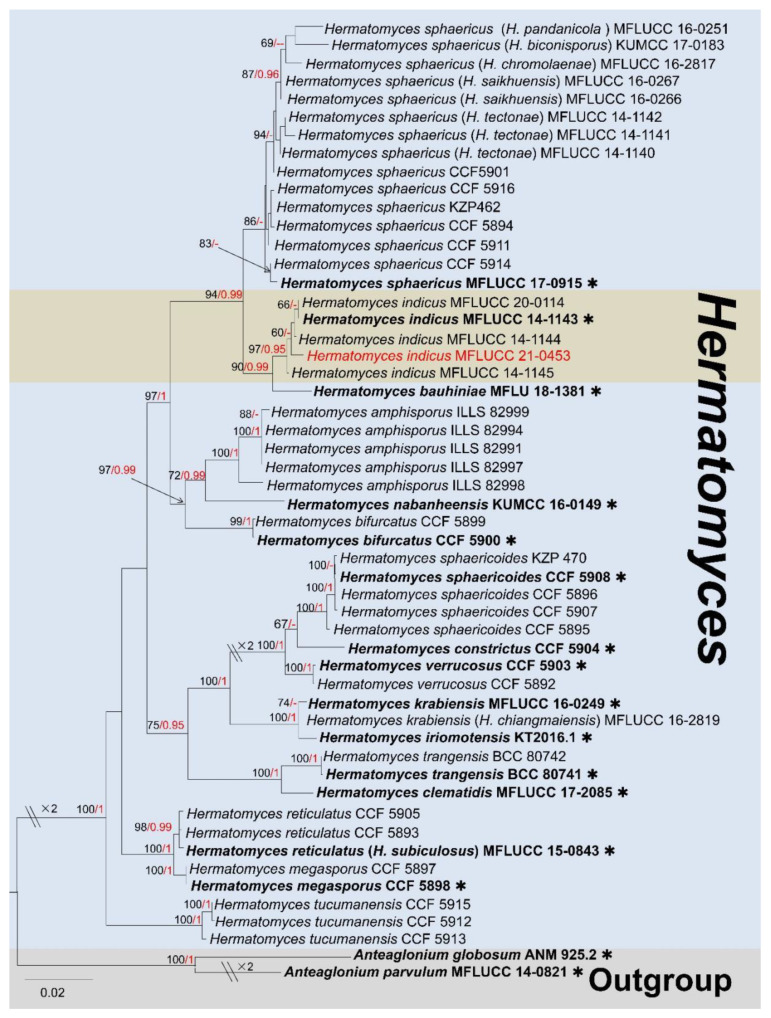
The phylogram based on a Maximum Likelihood analysis of combined LSU, ITS, *tef1*-*α* and *rpb2* sequence datasets. Related sequences were taken from Ren et al. [76]. The analyzed gene contains 53 fungal strains and 3650 total characters including gaps (LSU: 1–895 bp, *tef1*-*α*: 896–1874 bp, *rpb2*: 1875–2939 bp, ITS: 2940–3650 bp). The tree topology of the ML resembles BI. The matrix had distinct alignment patterns, with the final ML optimization likelihood value of −14,337.920997 (ln). All free model parameters were estimated by the RAxML model, with 1166 distinct alignment patterns and 26.63% of undetermined characters or gaps. Estimated base frequencies were as follows: A = 0.245211, C = 0.264483, G = 0.261115 and T = 0.229191, with substitution rates AC = 1.000809, AG = 3.682971, AT = 1.263474, CG = 0.858640, CT = 9.074183 and GT = 1.000000. The gamma distribution shape parameter alpha = 0.708454, and the Tree-Length = 0.779500. The final average standard deviation of split frequencies at the end of total MCMC generations was calculated as 0.009941 in BI analysis. The type strains are denoted in bold with the symbol “✱” at the ends, and newly introduced species in this study are denoted in red. The nodes provide bootstrap values of at least 60% (ML, left) and Bayesian posterior probabilities of at least 0.95 (BI, right); hyphens (-) signify values that are less than 60% in ML and less than 0.95 in BI. The bluish and pale brown backgrounds were used to distinguish *Hermatomyces* and the outgroup, while the brown background indicates the new isolate group.

Notes: Speggazini (1911) [72] established the genus *Hermatomyces* on the basis of material collected in Argentina, with *H. tucumanensis* as the type species. Speggazini [72] described only lenticular-type conidia and considered the cylindrical ones as conidiophores. However, with further morphological and phylogenetic investigations, the muriform, lenticular, hyaline or dematiaceous conidia of some *Hermatomyces* sp. were reported (previously referred to as monomorphic or dimorphic), and different species were mostly distinguished by cylindrical conidia [73,74]. Species of *Hermatomyces* seem to be limited by elevation and climate, and prefer to live on humid plant materials, including those found on immersed woody materials in water [73,75]. The ability of cylindrical conidia to germinate has not been proved; Koukol et al. [73] speculated that the function of cylindrical conidia is to support the lenticular conidia, and does not contribute to reproduction. To date, this genus includes a total of 34 records in Species Fungorum (2023) [58]. The phylogenetic placements of *Hermatomyces* species are shown in Figure 15.

**Figure 16:**
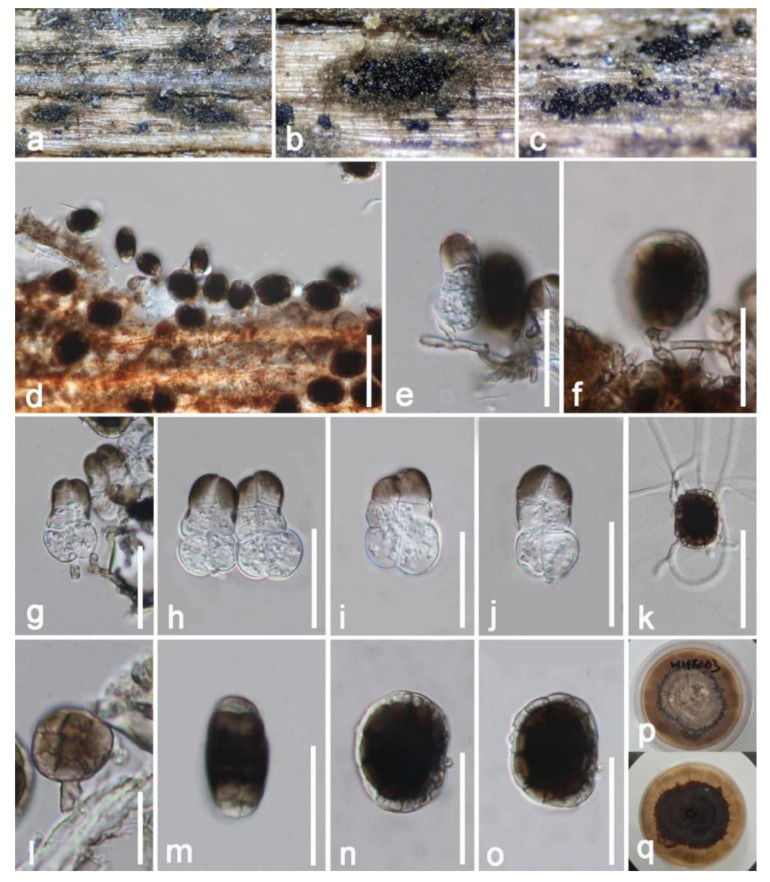
*Hermatomyces indicus* (HKAS 122676). (**a**–**c**) Colonies on the substrate; (**d**) colony on substrates observed under microscope; (**e**,**g**) conidiogenous cells bearing cylindrical conidia; (**f**,**l**) conidiogenous cells bearing lenticular conidia; (**h**–**j**) cylindrical conidia; (**k**) germinated lenticular conidium; (**m**–**o**) lenticular conidia; (**p**,**q**) colony on PDA. Scale bars: (**d**,**k**) = 50 μm; (**e**,**g**–**j**,**m**,**n**) = 30 μm; (**l**) = 5 μm.

*Hermatomyces indicus* Prasher & Sushma, Nova Hedwigia 99: 552 (2014) (Figure 16)

Index Fungorum Registration Identifier: IF805645

*Saprobic* on dead decaying branch of *Mangifera indica*. Sexual morph: Undetermined. Asexual morph: *Colonies* formed a dull zone on natural substrate, scattered to gregarious, in several groups, forming circular brownish mycelial outer region with abundantly sporulating center, with conidia easily liberated when touched with a needle. *Mycelium* 2–3 ($\bar{x}$ = 2.5 μm, n = 20) wide, superficial to immersed, effuse, brownish, comprising branched, septate, thick-walled hyphae. *Conidiophores* 3–6.5 μm ($\bar{x}$ = 6.5 μm, n = 20) high, 1–3 μm ($\bar{x}$ = 2 μm, n = 20) wide, micronematous, hyaline to brown, unbranched, straight or curved, erected from prostrate hyphae at the center of the circular colonies, thick walled, septate, smooth walled. *Conidiogenous cells* holoblastic, short, cylindrical, hyaline, thick walled, terminal. *Conidia* dimorphic; *Lenticular conidia* 30–35 × 20–25 µm ($\bar{x}$ = 32 × 22 µm, n = 20), turbinate, subglobose to ellipsoidal, comprising dark-brown cells in the center, subhyaline to pale brown in peripheral cells, in lateral view obovoid, dark-brown at the center, whitish to pale brown at lower and upper ends, sometimes carrying remnants of conidiogenous cells at base; *Cylindrical conidia* 30–35 × 15–21 µm ($\bar{x}$ = 32 × 18 µm, n = 20), with 2 rows of 3–4 cells in each column, broad at the lower cells, granulate, with lower cells usually hyaline, cylindrical, constricted at the septum, turbinate and dark brown at upper cells.

Culture characteristics: Conidia germinated within 8–12 h in PDA; colonies grew rapidly reaching around 40–50 mm after one month. Obverse: colonies circular, flat, radiate, coloration from hyaline turn to brown with the maturity, pale brown at the center, black-brown at the margin, clearly visible sparse mycelium extended. Reverse: deep brown in the middle area, brown near the margin, visible extended mycelium, without pigments produced in PDA.

Substratum: *Phoenix rupicola* [77]; *Tectona grandis* [78]; Decayed branch of *Mangifera indica* (this study).

Distribution: Chandigarh, India [77]; Chiang Rai Province, Thailand [78]; Yunnan Province, China (this study).

Material examined: China, Yunnan Province, Honghe, Menglong Village, on a dead and decaying branch of *Mangifera indica* (102°50′11′′ E, 23°41′01″ N, 500 m), 22 December 2020, E.F. Yang, HHE003 (HKAS 122676), living culture, KUMCC 21-0453. GenBank numbers: KUMCC 21-0453 = ITS: OQ379016, LSU: OQ379427, SSU: OQ372924, *tef1*-*α:* OQ379001, *rpb2*: OQ378994.

Notes: Our isolate largely matches *Hermatomyces indicus* (holotype: PAN 30900) based on morphological characteristics. Our strain and *Hermatomyces indicus* were comparable by two types of conidia. The lenticular conidia subglobose to ellipsoidal, with dark brown cells in the center, surrounded by pale peripheral cells at the periphery, with size (30–35 × 20–25 µm vs. 31 × 21.8 µm). Cylindrical conidia hyaline, with 2 rows of 3–4 cells in each column, brown at apex, becoming hyaline towards the lower side, constricted at the septum, containing numerous oil globules, with size (30–35 × 15–25 µm vs. 22–36 × 11–22 µm) [77]. Moreover, the BLASTn search results of ITS, LSU, *tef1*-*α* and *rpb2* showed our isolate (KUMCC 21-0453) is highly similar to *Hermatomyces indicus* strains (MFLUCC 14-1143, 14-1144, 14-1145) (>99%). The multi-gene phylogenetic trees indicated that our isolate clusters well with *Hermatomyces indicus* strains with high statistical support (Figure 15). Therefore, we report our isolate *Hermatomyces indicus* (KUMCC 21-0453) as a new host and geographic record from a decaying mango branch in China.

## 4. Discussion

To date, the family Plectosphaerellaceae includes more than 150 species in 24 genera following the publication “Outline of Fungi and fungus-like taxa—2021” [38]. Our isolate HKAS 122669 is relatively close to *Acremoniisimulans* (*Acremoniisimulans cocois* and *A. thailandensis)* with regard to the phylogenetic tree and sexual morphological characteristics [36,37]. In addition, BLASTn searches of SSU and LSU showed 99–100% similarity to *Acremoniisimulans* species; however, the BLASTn search results of ITS, *tef-α* and *rpb2* show extremely low similarity (=<91%) with *Acremoniisimulans* and other genera in the Plectosphaerellaceae. Therefore, the novel species *Acremoniisimulans honghensis* is established based on the differences in morphological features, phylogenetic trees and molecular data. The family Tubeufiaceae contains over 400 species in 47 genera [38]. The asexual morphology of *Excipulariopsis narsapurensis* was described by Spooner & Kirk and Pem et al. [39,40]. However, the precise phylogenetic position of *Excipulariopsis* has not yet been determined due to the absence of molecular data. Our study is the first to have solved this problem and uploaded the multiple genes to NCBI’s GenBank nucleotide database. The asexual morphologies of the genus *Delonicicola* are introduced herein for the first time. That will certainly help in updating our understanding of the morphology of both sexual and asexual characteristics of this taxon.

Taxonomic studies on saprobic or endophytic fungi associated with mango are still poorly conducted since most investigations have concentrated on pathogenic fungi associated with mango. However, the life modes and growth of fungi depend on a number of biotic (insect, bacterial and plant) and abiotic (habitat) factors [79,80,81,82]. Very few studies have reported saprobic fungi as a practically valuable group. For example, Botrel et al. [83] reported that saprobic *Phialomyces macrosporus* has the potential to be employed in the management of coffee halo blight, which caused by *Pseudomonas syringae pv. garcae*, Monkai et al. [84] isolated four *Cytospora* species from decaying leaves, and three of them (*Cytospora shoreae*, *C. phitsanulokensis* and *C. chiangmaiensis*) showed 60–75% growth inhibition against pathogens *Colletotrichum viniferum* and *Fusarium sambucinum* in vitro. Therefore, deep studies of saprobic fungi are necessary, especially secondary metabolites of saprobic fungi and their life mode changes. During our investigations of microfungi associated with mango in Yunnan Province (China), we observed a pattern of high fungal diversity in the mango ([16,17], in this study). Of the 28 species of fungi introduced, based on morphology and phylogeny, most belong to the order Pleosporales (11 species, 39%), with four species belonging to the order Xylariales and three species to the order Botryosphaeriales. Together with two genera (*Mangifericola* and *Mangifericomes*) and 11 new species so far established, there is certainly an incredible diversity of new species—39% (Table 2). Among these, the species from the Botryosphaeriales, Pleosporales and Xylariales are dominant saprobic fungi on mango substrates. The results of this study also suggest that numerous additional new and interesting species of fungi are likely to be discovered in the future on mango substrates.

## Figures and Tables

**Table 1:** The PCR conditions and primers used in this study.

Locus	Definition	Primer	Annealing (PCR)	Reference
ITS	Internal transcribed spacer	ITS4	^a^ 55 °C, 50 s ^b^	[20]
		ITS5		
LSU	Large subunit 28S	LR0R	^a^ 55 °C, 50 s ^b^	[21]
		LR5		
*rpb2*	DNA-dependent RNA polymerase II largest subunit	5F	^a^ 57 °C, 55 s ^b^	[22]
		7cR		
SSU	The partial18S small subunit	NS1	^a^ 55 °C, 50 s ^b^	[20]
		NS4		
*tef1*-*α*	Translation elongation factor 1 alpha	983F	^a^ 56 °C, 50 s ^b^	[23]
		2218R		
*tub2*	Beta-tubulin 2	Bt2a	^a^ 52 °C, 50 s ^b^	[24]
		Bt2b		

^a^ Initial denaturation of 2 min at 94 °C, followed by 35 cycles of denaturation at 95 °C for 30 s, annealing temperature and time for different genes are mentioned in Table 1. ^b^ 90 s of extension at 72 °C and a final extension of 10 min at 72 °C.

**Table 2:** List of saprobic fungi associated with mango from Honghe and Baoshan, Yunnan, China (Yang et al. [16,17] and this study); the bold text indicates new species.

Class	Order	Family	Species	Herbarium No.
Dothideomycetes	Botryosphaeriales	Aplosporellaceae	*Aplosporellaartocarpi*	HKAS 122656
		Botryosphaeriaceae	*Lasiodiplodia theobromae*	HKAS 122660
		Botryosphaeriaceae	*Lasiodiplodiapseudotheobromae*	HKAS 122658
	Monoblastiales	Monoblastiaceae	*Heleiosabarbatula*	HKAS 122668
	Pleosporales	Hermatomycetaceae	*Hermatomyces indicus*	HKAS 122676
		*Incertaesedis*	*Crassipariesquadrisporus*	HKAS 122192
		*Incertaesedis*	***Mangifericomes hongheensis***	HKAS 122188
		Massarinaceae	*Vaginatisporaamygdali*	HKAS 122195
		Melanommataceae	*Byssosphaeriasiamensis*	HKAS 122197
		Neomassariaceae	***Neomassaria hongheensis***	HKAS 122191
		Paradictyoarthriniaceae	*Paradictyoarthriniumdiffractum*	HKAS 122194
		Parabambusicolaceae	***Paramonodictys hongheensis***	HKAS 122190
		Parabambusicolaceae	***Paramonodictys yunnanensis***	HKAS 122189
		Phaeoseptaceae	*Phaeoseptummali*	HKAS 122193
		Torulaceae	*Torulafici*	HKAS 122196
	Tubeufiales	Tubeufiaceae	*Excipulariopsisnarsapurensis*	HKAS 122680
Eurotiomycete	Chaetothyriales	Cyphellophoraceae	***Cyphellophora hongheensis***	HKAS 122661
	Mycocaliciales	Mycocaliciaceae	***Chaenothecopsis hongheensis***	HKAS 122672
Sordariomycetes	Calosphaeriales	Pleurostomataceae	*Pleurostomaootheca*	HKAS 122679
	Coronophorales	Nitschkiaceae	*Fracchiaeamyricoides*	HKAS 122671
	Delonicicolales	Delonicicolaceae	*Delonicicolasiamense*	HKAS 122662
	Diaporthales	Diaporthaceae	***Diaporthe hongheensis***	HKAS 122657
	Glomerellales	Plectosphaerellaceae	***Acremoniisimulans hongheensis***	HKAS 122669
	Sordariales	Helminthosphaeriaceae	***Hilberina hongheensis***	HKAS 122677
	Xylariales	Diatrypaceae	***Mangifericola hongheensis***	HKAS 122665
		Diatrypaceae	*Paraeutypellacitricola*	HKAS 122667
		Hypoxylaceae	***Hypoxylon hongheensis***	HKAS 122663
		Hypoxylaceae	*Hypomontagnellamonticulosa*	HKAS 122664

## Data Availability

Not applicable.

## Supplementary Materials

The following supporting information can be downloaded at: https://www.mdpi.com/article/10.3390/jof9060680/s1, Tables S1–S8: The names, isolate numbers, and corresponding GenBank accession numbers of the taxa used in phylogenetic trees; Supplementary information (S1–S8): the detail information of phylogenetic trees.
