# Supplementary material for: Taxonomic and Phylogenetic Studies of Saprobic Fungi Associated with Mangifera indica in Yunnan, China
Source: J Fungi (Basel). 2023 Jun 16;9(6):680. doi: 10.3390/jof9060680 (PMC10301488; doi:10.3390/jof9060680)
Supplement: Supplementary file 1 [file jof-09-00680-s001.zip › jof-2388040-supplementary.pdf]

Supplementary materials

Table S1. The names, isolate numbers, and corresponding GenBank accession numbers of the taxa used in Figure 1. The taxa produced in this study are indicated in red, and the type strains are indicated in bold with "✱".

Species	Isolate No.	GenBank accession No.			
		LSU	ITS	*tef1-α*	*rpb2*
Acremoniisimulans hongheensis	**HKAS 122669 ✱**	**OQ379416**	**OQ379005**	**OQ378995**	**OQ378988**
Acremoniisimulans hongheensis	HKAS 122670	OQ379417	OQ379006	OQ378996	OQ378989
Acremoniisimulans thailandensis	**MFLU 18-0012 ✱**	**MH260315**	**MH275081**	/	/
Acremonjisimulans cocois	**MFLUCC 15-0817 ✱**	**ON650672**	**ON650683**	/	/
Acrostalagmus annulatus	CBS 121.84	LR025802	LR026673	LR026374	LR026104
Acrostalagmus annulatus	CBS 185.70	LR025803	LR026674	LR026375	LR026105
Acrostalagmus annulatus	CBS 450.85	LR025804	LR026675	LR026376	LR026106
Acrostalagmus luteoalbus	CBS 112.16	LR025797	LR026668	LR026369	LR026101
Acrostalagmus luteoalbus	CBS 222.60	LR025794	LR026665	LR026366	LR026099
Acrostalagmus luteoalbus	CBS 236.55	LR025798	LR026669	LR026370	LR026102
Brunneochlamydosporium cibotii	**CBS 109240 ✱**	**LR025807**	**LR026678**	**LR026380**	/
Brunneochlamydosporium macroclavatum	CBS 373.93	LR025809	LR026680	LR026382	LR026109
Brunneochlamydosporium macroclavatum	CBS 823.73	LR025810	LR026681	LR026383	LR026110
Brunneochlamydosporium macroclavatum	CBS 101249	LR025811	LR026682	LR026384	/
Brunneochlamydosporium nepalense	**CBS 971.72 ✱**	**LR025813**	**LR026684**	**LR026386**	**LR026112**
Brunneochlamydosporium nepalense	CBS 112045	LR025814	LR026685	LR026387	LR026113
Brunneochlamydosporium terrestre	CBS 112777	LR025819	LR026690	LR026392	LR026118
Brunneomyces brunnescens	**CBS 559.73 ✱**	**HQ231966**	**LN810520**	**LN810534**	**LR026119**

Brunneomyces europaeus	CBS 560.86	LN810511	LN810518	LN810537	LN810527
Brunneomyces europaeus	**CBS 652.96 ✱**	**LN810512**	**LN810519**	**LN810538**	**LN810528**
Brunneomyces hominis	**FMR 10429 ✱**	**LN810509**	**KP131517**	**LN810535**	**/**
Chlamydosporiella restricta	CBS 119.97	LR025820	LR026691	LR026393	LR026120
Chlamydosporiella restricta	CBS 177.40	LR025821	LR026692	LR026394	LR026121
Chlamydosporiella restricta	**CBS 178.40 ✱**	**LR025822**	**LR026693**	**LR026395**	**LR026122**
Chlamydosporiella restricta	CBS 434.83	LR025823	LR026694	LR026396	LR026123
Chlamydosporiella restricta	CBS 443.66	LR025824	LR026695	LR026397	LR026124
Chlamydosporiella restricta	CBS 716.88	LR025825	LR026696	LR026398	LR026125
Chlamydosporiella restricta	CBS 988.69	LR025826	LR026697	LR026399	/
Chordomyces albus	**CBS 987.87 ✱**	**JX158444**	**DQ825970**	**JX158400**	**JX158466**
Chordomyces albus	CBS 206.70	LR025829	LR026700	LR026402	LR026128
Chordomyces albus	CBS 299.70E	LR025830	LR026701	LR026403	LR026129
Chordomyces albus	CBS 204.70	LR025827	LR026698	LR026400	LR026126
Chordomyces albus	CBS 205.70	LR025828	LR026699	LR026401	LR026127
Chordomyces antarcticus	CBS 120042	KJ443108	KJ443240	KJ443196	KJ443156
Chordomyces antarcticus	**CBS 120045 ✱**	KJ443109	KJ443241	KJ443197	KJ443157
Chordomyces antarcticus	CBS 120046	KJ443110	KJ443242	KJ443198	KJ443158
Furcasterigmium furcatum	CBS 116548	LR025842	LR026712	LR026409	LR026134
Furcasterigmium furcatum	CBS 116550	LR025843	LR026713	LR026410	LR026135
Furcasterigmium furcatum	**CBS 122.42 ✱**	**LR025838**	**LR026709**	**LR026408**	**LR026133**
Fuscohypha expansa	CBS 103.95	LR025844	LR026714	LR026411	/
Fuscohypha expansa	**CBS 418.89 ✱**	**LR025845**	**LR026715**	**LR026412**	**LR026136**
Gibellulopsis aquatica	**CBS 117131 ✱**	**LR025850**	**LR026720**	**LR026414**	**/**
Gibellulopsis capsici	CBS 175.75	LR025871	LR026741	LR026432	LR026152

Gibellulopsis capsici	**CBS 290.30 ✱**	**LR025872**	**LR026742**	**LR026433**	**/**
Gibellulopsis capsici	CBS 345.39	LR025873	LR026743	LR026434	LR026153
Gibellulopsis capsici	CBS 565.78C	LR025884	LR026754	LR026444	LR026158
Gibellulopsis capsici	CBS 892.70	LR025885	LR026755	LR026445	LR026159
Gibellulopsis capsici	CBS 100826	LR025886	LR026756	LR026446	LR026160
Gibellulopsis capsici	CBS 100827	LR025887	LR026757	LR026447	LR026161
Gibellulopsis catenata	**CBS 113951 ✱**	**LR025851**	**LR026721**	**LR026415**	**LR026137**
Gibellulopsis fusca	CBS 308.38	LR025852	LR026722	LR026416	LR026138
Gibellulopsis fusca	CBS 402.80	LR025853	LR026723	LR026417	LR026139
Gibellulopsis fusca	**CBS 560.65 ✱**	**LR025854**	**LR026724**	**LR026418**	**LR026140**
Gibellulopsis nigrescens	**CBS 120949✱**	**NG_067330**	**NR_149327**	**LR026429**	**LR026149**
Gibellulopsis nigrescens	CBS 123176	LR025869	LR026739	LR026430	LR026150
Lectera capsici	**CBS 142534 ✱**	**KY979825**	**KY979770**	**LR026454**	**LR026166**
Lectera colletotrichoides	IMI 303685	LR025894	JQ647450	LR026455	LR026167
Lectera colletotrichoides	IMI 332702	LR025895	JQ647428	LR026456	LR026168
Lectera colletotrichoides	IMI 265740	LR025896	JQ647449	LR026457	LR026169
Lectera longa	**IMI 181698 ✱**	**LR025897**	**JQ647448**	**LR026458**	**LR026170**
Lectera phaseoli	IMI 366179	LR025898	JQ693168	LR026459	LR026171
Longitudinalis nabanheensis	**KUMCC 16-0145 ✱**	**NG_068250**	**KY882037**	**KY882040**	**/**
Monilochaetes infuscans	CBS 379.77	GU180645	LR026764	LR026460	GU180658
Monilochaetes infuscans	**CBS 869.96 ✱**	**GU180639**	**GU180626**	**LR026461**	**GU180657**
Musicillium elettariae	**CBS 252.80 ✱**	**LR025899**	**LR026765**	**LR026462**	**LR026172**
Musicillium elettariae	CBS 110322	LR025900	LR026766	LR026463	/
Musicillium elettariae	CBS 140681	LR025901	LR026767	LR026464	LR026173
Musicillium theobromae	CBS 360.76	LR025904	LR026770	LR026465	LR026175

Musicillium theobromae	CBS 385.32	LR025905	LR026771	LR026466	LR026176
Musicillium theobromae	CBS 397.58	LR025906	LR026772	LR026467	LR026177
Musicillium tropicale	CBS 458.51	LR025915	LR026781	LR026475	LR026184
Musicillium tropicale	CBS 100951	LR025916	LR026782	LR026476	LR026185
Musicillium tropicale	**CBS 120009 ✱**	**LR025917**	**LR026783**	**LR026477**	**LR026186**
Musidium stromaticum	**CBS 863.73 ✱**	**HQ232143**	**DQ825969**	**LN810533**	**/**
Musidium stromaticum	CBS 132.74	LR025919	LR026785	LR026479	LR026187
Musidium stromaticum	CBS 133.74	LR025920	LR026786	LR026480	LR026188
Musidium stromaticum	CBS 135.74A	LR025922	LR026787	LR026482	LR026189
Nigrocephalum collariferum	CBS 124585	LR025928	FJ765365	LR026485	LR026192
Nigrocephalum collariferum	**CBS 124586 ✱**	**LR025929**	**FJ765367**	**LR026486**	**LR026193**
Paragibellulopsis chrysanthemi	**MAFF 242621 ✱**	**KC287230**	**KC287235**	**KC287232**	**/**
Paragibellulopsis chrysanthemi	MAFF 243429	KC287229	KC287234	KC287231	/
Paragibellulopsis chrysanthemi	MAFF 243430	KC287228	KC287233	/	/
Paramusicillium asperulatum	**CBS 120158 ✱**	**LR025930**	**LR026792**	**LR026487**	**LR026194**
Phialoparvum bifurcatum	**CBS 299.70B ✱**	**LR025931**	**LR026793**	**LR026488**	**LR026195**
Plectosphaerella alismatis	CBS 113362	LR025932	LR026794	LR026489	LR026196
Plectosphaerella citrullae	CBS 131740	LR025933	LR026795	LR026490	/
Plectosphaerella citrullae	**CBS 131741 ✱**	**LR025934**	**LR026796**	**LR026491**	**LR026197**
Plectosphaerella cucumerina	**CBS 137.37 ✱**	**LR025936**	**LR026798**	**LR026493**	**LR026199**
Plectosphaerella cucumerina	CBS 139.60	LR025937	LR026799	LR026494	LR026200
Plectosphaerella cucumerina	CBS 286.64	LR025938	LR026800	LR026495	LR026201
Plectosphaerella delsorboi	**CBS 116708 ✱**	**LR025948**	**LR026810**	**LR026505**	**LR026209**
Plectosphaerella humicola	**CBS 423.66 ✱**	**LR025949**	**LR026811**	**LR026506**	**LR026210**
Plectosphaerella melonis	**CBS 489.96T ✱**	**LR025950**	**LR026812**	**LR026507**	**/**

Plectosphaerella oligotrophica	CBS 440.90	LR025952	LR026814	LR026509	LR026211
Plectosphaerella pauciseptata	CBS 131745	LR025954	LR026816	LR026511	LR026212
Plectosphaerella plurivora	**CBS 131742 ✱**	**LR025967**	**LR026829**	**LR026524**	**LR026219**
Plectosphaerella populi	**CBS 139623 ✱**	**KR476783**	**KR476750**	**LR026527**	**LR026222**
Plectosphaerella ramiseptata	**CBS 131861 ✱**	**LR025970**	**LR026832**	**LR026530**	**LR026225**
Plectosphaerella sinensis	**ACCC 39145 ✱**	**KX527891**	**KX527888**	**/**	**/**
Sayamraella subula	**BCC 78964 ✱**	**LR025971**	**LR026833**	**LR026531**	**LR026226**
Sodiomyces alcalophilus	**CBS 114.92 ✱**	**JX158443**	**JX158421**	**JX158399**	**JX158465**
Sodiomyces alkalinus	**CBS 110278 ✱**	**JX158427**	**NR_145378**	**JX158383**	**JX158449**
Sodiomyces magadii	**CBS 137619 ✱**	**KJ443148**	**KJ443278**	**/**	**/**
Sodiomyces tronii	**CBS 137618 ✱**	**KJ443147**	**KJ443277**	**/**	**/**
Stachylidium bicolor	**CBS 121802 ✱**	**LR025972**	**LR026834**	**LR026532**	**/**
Stachylidium pallidum	BCC 79031	LR025973	LR026835	LR026533	LR026227
Summerbellia oligotrophica	CBS 299.70G	LR025846	LR026716	LR026413	/
Theobromium fuscum	**CBS 112271 ✱**	**LR025976**	**LR026839**	**LR026535**	**LR026229**
Verticillium albo-atrum	CBS 388.82	LR025978	LR026841	LR026537	LR026231
Verticillium albo-atrum	**CBS 130340 ✱**	**LR025984**	**LR026847**	**LR026543**	**LR026233**
Verticillium alfalfae	**CBS 130603 ✱**	**LR025988**	**LR026851**	**LR026547**	**LR026236**
Verticillium dahliae	CBS 179.66	LR025992	LR026854	LR026549	LR026238
Verticillium isaacii	CBS 100839	LR026034	LR026895	LR026575	LR026258
Verticillium isaacii	**CBS 130343 ✱**	**LR026038**	**LR026899**	**LR026577**	**/**
Verticillium klebahnii	**CBS 130344 ✱**	**LR026039**	**LR026900**	**LR026578**	**/**
Verticillium longisporum	**CBS 124.64 ✱**	**LR026040**	**LR026901**	**/**	**/**
Verticillium nonalfalfae	**CBS 130339 ✱**	**LR026074**	**LR026935**	**LR026590**	**/**
Verticillium nonalfalfae	CBS 454.51	LR026070	LR026931	LR026586	LR026279

Verticillium nubilum	**CBS 457.51 ✱**	**LR026076**	**LR026937**	**LR026591**	**LR026282**
Verticillium tricorpus	**CBS 447.54 ✱**	**LR026083**	**LR026944**	**/**	**/**
Verticillium tricorpus	CBS 545.79	LR026084	LR026945	LR026597	LR026284
Verticillium tricorpus	CBS 803.97	LR026085	LR026946	LR026598	LR026285
Verticillium zaregamsianum	**CBS 130342 ✱**	**LR026098**	**LR026959**	**LR026610**	**/**
Verticillium zaregamsianum	CBS 100842	LR026097	LR026958	LR026609	/
Xenoplectosphaerella clematidis	**MFLUCC 17-2067 ✱**	**NG_071256**	**NR_172181**	**MT394674**	**MT394722**

Table S2. The names, isolate numbers, and corresponding GenBank accession numbers of the taxa used in Figure 3. The taxa produced in this study are indicated in red, and the type strains are indicated in bold with "✱".

Species	Strain	GenBank accession No.			
		ITS	LSU	***tef1-a***	***rpb2***
Acanthohelicospora aurea	GZCC 16-0059	KY321322	KY321325	KY792599	MF589910
Acanthohelicospora scopula	MFLUCC10-0116	KF301526	KF301534	KF301555	/
Acanthohelicospora scopulum	ANM 386	GQ856141	GQ850489	/	/
Acanthohelicospora scopulum	ANM 95	GQ856142	GQ850490	/	/
Acanthostigma chiangmaiensis	**MFLUCC10-0125 ✱**	**JN865209**	**JN865197**	**/**	**/**
Acanthostigma perpusillum	UAMH 7237	AY916492	AY856892	/	/
Acanthostigmina minutum	ANM 818	/	GQ850488	/	/
Acanthostigmina minutum	ANM 238	/	GQ850486	/	/
Acanthostigmina multiseptatum	ANM 665	GQ856144	GQ850493	/	/

Acanthostigmina multiseptatum	**ANM 475 ✱**	**GQ856145**	**GQ850492**	**/**	**/**
Acanthotubeufia filiformis	ANM 101	/	GQ850495	/	/
Acanthotubeufia filiformis	ANM 514	GQ856146	GQ850494	/	/
Aquaphila albicans	BCC 3520	DQ341098	DQ341102	/	/
Aquaphila albicans	BCC 3543	DQ341096	DQ341101	/	/
Berkleasmium aquaticum	MFLUCC 17-0039	KY790443	KY790431	KY792607	MF535267
Berkleasmium aquaticum	GZCC 16-0056	KY790442	KY790430	KY792606	MF535266
Berkleasmium aquaticum	**MFLUCC 17-0049 ✱**	**KY790444**	**KY790432**	**KY792608**	**MF535268**
Berkleasmium brunneisporum	MFLUCC 16-1127	KY790447	KY790435	KY792611	MF535269
Berkleasmium brunneisporum	**MFLUCC 16-0015 ✱**	**KY790446**	**KY790434**	**KY792610**	**/**
Berkleasmium concinnum	DAOM:696499	KY611397	KY611399	/	/
Berkleasmium fusiforme	MFLUCC 17-1988	MH558696	MH558823	MH550887	MH551010
Berkleasmium guangxiense	**MFLUCC 17-0042 ✱**	**KY790448**	**KY790436**	**KY792612**	**MF535270**
Berkleasmium latisporum	**MFLUCC 16-0019 ✱**	**KY790449**	**KY790437**	**KY792613**	**MF535271**
Berkleasmium longisporum	MFLUCC 17-2002	MH558699	MH558826	MH550890	MH551013
Berkleasmium longisporum	**MFLUCC 17-1999 ✱**	**MH558698**	**MH558825**	**MH550889**	**MH551012**
Berkleasmium thailandicum	MFLUCC 17-1984	MH558700	MH558827	MH550891	MH551015
Berkleasmium thailandicum	MFLUCC 17-2000	MH558701	MH558828	MH550892	MH551016
Boerlagiomyces macrospora	MFLUCC 12-0388	KU144927	KU764712	KU872750	/
Botryosphaeria pseudoramosa	**CERC 2001 ✱**	**KX277989**	**MF410031**	**/**	**MF410140**
Botryosphaeria wangensis	**CERC 2298 ✱**	**KX278002**	**MF410044**	**/**	**MF410153**
Camporesiomyces mali	**KUMCC 19-0216 ✱**	**MN792813**	**MN792811**	**MN794018**	**/**
Camporesiomyces patagoniensis	**BBB:MVB 573 ✱**	**NG_056997**	**JN127359**	**/**	**/**
Camporesiomyces vaccinii	**CBS:216.90 ✱**	**MH862204**	**MH873889**	**/**	**/**
Camporesiomyces vaccinii	CBS 216.91	AY916486	AY856879	/	/

Chlamydotubeufia cylindrica	**MFLU 17-1177 ✱**	**NR_159761**	**NG_066230**	**MH550893**	**MH551018**
Chlamydotubeufia huaikangplaensis	MFLUCC 16-0227	KY678765	KY678757	KY792596	MF535260
Chlamydotubeufia huaikangplaensis	MFLUCC 16-0023	KY678766	KY678758	KY792597	MF535259
Chlamydotubeufia krabiensis	**MFLUCC 16-1134 ✱**	**KY678767**	**KY678759**	**KY792598**	**MF535261**
Dematiohelicoma pulchrum	MUCL 39827	AY916457	AY856872	/	/
Dematiohelicomyces helicosporus	MFLUCC 16-0007	MH558704	MH558832	MH550895	MH551020
Dematiohelicomyces helicosporus	MFLUCC 16-0003	MH558703	MH558831	MH550894	MH551019
Dematiohelicosporum guttulatum	**MFLUCC 17-2011 ✱**	**MH558705**	**MH558833**	**MH550896**	**MH551021**
Dematiotubeufia chiangraiensis	MFLU 10–0048	/	/	KF301551	/
Dictyospora thailandica	**MFLUCC 16-0001 ✱**	**KY873627**	**KY873622**	**KY873286**	**MH551023**
Dictyospora thailandica	MFLUCC 18-0641	MH558706	MH558834	MH550897	MH551022
Discotubeufia browneae	**MFLUCC 17-0908 ✱**	**NR_163321**	**MK347938**	**/**	**MK434903**
Excipulariopsis narsapurensis	KUMCC 21-0464	OQ379007	OQ379418	OQ378997	OQ378990
Excipulariopsis narsapurensis	KUMCC 21-0465	OQ379008	OQ379419	OQ378998	OQ378991
Helicangiospora lignicola	MFLUCC 11–0378	KF301523	KF301531	KF301552	/
Helicoarctatus aquaticus	**MFLUCC 17-1996 ✱**	**MH558707**	**MH558835**	**MH550898**	**MH551024**
Helicodochium aquaticum	**MFLUCC 17-2016 ✱**	**MH558709**	**MH558837**	**MH550900**	**MH551026**
Helicodochium aquaticum	MFLUCC 18-0490	MH558710	MH558838	MH550901	MH551027
Helicohyalinum aquaticum	MFLUCC 16-0014	MH558711	MH558839	MH550902	MH551028
Helicohyalinum aquaticum	**MFLUCC 16-1131 ✱**	**KY873625**	**NG_070412**	**KY873284**	**MF535257**
Helicohyalinum infundibulum	**MFLUCC 16-1133 ✱**	**MH558712**	**MH558840**	**MH550903**	**MH551029**
Helicoma aquaticum	**MFLUCC 17-2025 ✱**	**MH558713**	**MH558841**	**MH550904**	**MH551030**
Helicoma brunneisporum	**MFLUCC 17-1983 ✱**	**MH558714**	**MH558842**	**MH550905**	**MH551031**
Helicoma guttulatum	MFLUCC 16-0022	KX454171	KX454172	MF535254	MH551032
Helicoma hongkongense	**MFLUCC 17-2005 ✱**	**MH558716**	**MH558843**	**MH550907**	**MH551033**

Helicoma longisporum	MFLUCC 16-0211	MH558719	MH558845	MH550910	MH551036
Helicoma nematosporum	MFLUCC 16-0011	MH558722	MH558848	MH550913	MH551039
Helicoma rubriappendiculatum	**MFLUCC 18-0491 ✱**	**MH558723**	**MH558849**	**MH550914**	**MH551040**
Helicoma rufum	**MFLUCC 17-1806 ✱**	**MH558724**	**MH558850**	**MH550915**	**/**
Helicoma septoconstrictum	**MFLUCC 17-2001 ✱**	**MH558726**	**MH558852**	**MH550917**	**MH551042**
Helicoma septoconstrictum	MFLUCC 17-1991	MH558725	MH558851	MH550916	MH551041
Helicoma siamense	MFLUCC 12-0563	KU144928	KU764713	KU872751	/
Helicomyces colligatus	**MFLUCC 16-1132 ✱**	**MH558727**	**MH558853**	**MH550918**	**MH551043**
Helicomyces hyalosporus	**MFLUCC 17-0051 ✱**	**MH558731**	**MH558857**	**MH550922**	**MH551047**
Helicomyces torquatus	**MFLUCC 16-0217 ✱**	**MH558732**	**MH558858**	**MH550923**	**MH551048**
Helicosporium aquaticum	**MFLUCC 17-2008 ✱**	**NR_160371**	**/**	**MH550924**	**MH551049**
Helicosporium flavisporum	**MFLUCC 17-2020 ✱**	**MH558734**	**MH558860**	**MH550925**	**MH551050**
Helicosporium flavum	**MFLUCC 16-1230 ✱**	**KY873626**	**KY873621**	**KY873285**	**/**
Helicosporium gracile	CBS 284.54	MH857334	MH868875	/	/
Helicosporium luteosporum	**MFLUCC 16-0226 ✱**	**KY321324**	**KY321327**	**KY792601**	**MH551056**
Helicosporium setiferum	**MFLUCC 17-1994 ✱**	**MH558735**	**MH558861**	**MH550926**	**MH551051**
Helicosporium setiferum	MFLUCC 17-2007	MH558737	MH558863	MH550928	MH551053
Helicosporium sp.	NBRC 9014	AY916489	AY856903	/	/
Helicosporium vegetum	CBS 941.72	AY916488	AY856883	/	/
Helicosporium vesicarium	MFLUCC 17-1795	MH558739	MH558864	MH550930	MH551055
Helicosporium viridiflavum	**MFLUCC 17-2336 ✱**	**MH558738**	**/**	**MH550929**	**MH551054**
Helicotruncatum palmigenum	MFLUCC 15-0993	MT627685	MN913690	/	/
Helicotubeufia guangxiensis	**MFLUCC 17-0040 ✱**	**MH290018**	**MH290023**	**MH290028**	**MH290033**
Helicotubeufia hydei	**MFLUCC 17-1980 ✱**	**MH290021**	**MH290026**	**MH290031**	**MH290036**
Helicotubeufia jonesii	**MFLUCC 17-0043 ✱**	**MH290020**	**MH290025**	**MH290030**	**MH290035**

Kamalomyces mangrovei	MFLUCC 17-0407	MN047097	MN017863	MN077072	/
Kamalomyces thailandicus	**MFLUCC 13-0233 ✱**	**MF506884**	**MF506882**	**MF506886**	**/**
Kamalomyces thailandicus	MFLUCC 11-0158	MF506883	MF506881	MF506885	MF506887
Kevinhydea brevistipitata	MFLUCC 18–1269	MH747102	MH747115	/	/
Manoharachariella tectonae	MFLUCC 12-0170	KU144935	KU764705	KU872762	/
Muripulchra aquatica	**KUMCC 15-0276 ✱**	**KY320534**	**KY320551**	**KY320564**	**MH551058**
Muripulchra aquatica	**KUMCC 15-0245 ✱**	**KY320533**	**KY320550**	**KY320563**	**MH551057**
Neoacanthostigma fusiforme	MFLUCC 11–0510	KF301529	KF301537	/	/
Neochlamydotubeufia fusiformis	**MFLUCC 16-0016 ✱**	**MH558740**	**MH558865**	**MH550931**	**MH551059**
Neochlamydotubeufia khunkornensis	MFLUCC 16-1126	MH558743	MH558868	MH550934	MH551062
Neohelicoma fagacearum	MFLUCC110379	KF301524	KF301532	KF301553	/
Neohelicomyces aquaticus	KUMCC 15-0463	KY320529	KY320546	KY320562	MH551065
Neohelicomyces aquaticus	**MFLUCC 16-0993 ✱**	**KY320528**	**KY320545**	**KY320561**	**MH551066**
Neohelicomyces dehongensis	**MFLUCC 18-1029 ✱**	**MT627701**	**MN913709**	**MT954393**	**/**
Neohelicomyces hyalosporus	**GZCC 16-0086 ✱**	**MH558745**	**MH558870**	**MH550936**	**MH551064**
Neohelicosporium acrogenisporum	**MFLUCC 17-2019 ✱**	**MH558746**	**MH558871**	**MH550937**	**MH551069**
Neohelicosporium aquaticum	MFLUCC 17-1519	MF467916	MF467929	MF535242	MF535272
Neohelicosporium astrictum	**MFLUCC 17-2004 ✱**	**MH558747**	**MH558872**	**MH550938**	**MH551070**
Neohelicosporium ellipsoideum	**MFLUCC 16-0229 ✱**	**MH558748**	**MH558873**	**MH550939**	**MH551071**
Neohelicosporium fusisporum	MFUCC 16-0642	MG017612	MG017613	MG017614	/
Neohelicosporium guangxiense	GZCC 16-0068	MH558749	MH558874	MH550940	MH551072
Neohelicosporium guangxiense	MFLUCC 17-0054	MH558750	MH558875	MH550941	MH551073
Neohelicosporium hyalosporum	**GZCC 16-0076 ✱**	**MF467923**	**MF467936**	**MF535249**	**MF535279**
Neohelicosporium irregulare	**MFLUCC 17-1796 ✱**	**MH558752**	**MH558877**	**MH550943**	**MH551075**
Neohelicosporium krabiense	**MFLUCC 16-0224 ✱**	**MH558754**	**MH558879**	**MH550945**	**MH551077**

Neohelicosporium laxisporum	**MFLUCC 17-2027 ✱**	**MH558755**	**MH558880**	**MH550946**	**MH551078**
Neohelicosporium ovoideum	**GZCC 16-0064 ✱**	**MH558756**	**MH558881**	**MH550947**	**MH551079**
Neohelicosporium parvisporum	MFLUCC 16-0218	MF467927	MF467940	MH550955	MH551087
Neohelicosporium parvisporum	MFLUCC 17-1521	MH558758	MH558883	MH550949	MH551081
Neohelicosporium parvisporum	MFLUCC 17-1804	MH558760	MH558885	MH550951	MH551083
Neohelicosporium thailandicum	**MFLUCC 16-0221 ✱**	**MF467928**	**MF467941**	**MF535253**	**MF535283**
Neotubeufia krabiensis	**MFLUCC 16-1125 ✱**	**MG012031**	**MG012024**	**MG012010**	**MG012017**
Parahelicomyces paludosus	AR 4206	DQ341095	DQ341103	/	/
Parahelicomyces quercus	**MFUCC 17-0895 ✱**	**MK347720**	**MK347934**	**MK360077**	**MK434906**
Pleurohelicosporium parvisporum	**MFLU 17-1124 ✱**	**MH558764**	**MH558889**	**MH550956**	**MH551088**
Podonectria kuwanaspidis	SICAUCC 21-0007	MW484994	MW462905	MW462116	MW462123
Podonectria kuwanaspidis	**SICAUCC 21-0002 ✱**	**MW484989**	**MW462900**	**MW462112**	**MW462119**
Podonectria novae-zelandiae	SICAUCC 21-0005	MW484992	MW462903	MW462115	MW462122
Podonectria sichuanensis	SICAUCC 21-0001	MW484988	MW462899	MW462111	MW462118
Pseudohelicomyces aquaticus	**MFLUCC 16-0234 ✱**	**MH558766**	**MH558891**	**MH550958**	**MH551092**
Pseudohelicomyces talbotii	**MFLUCC 17-2021 ✱**	**MH558765**	**MH558890**	**MH550957**	**MH551091**
Pseudohelicoon gigantisporum	BCC 3550	AY916467	AY856904	/	/
Tamhinispora indica	**NFCCI 2924 ✱**	**KC469282**	**KC469283**	**/**	**/**
Tamhinispora srinivasanii	NFCCI 4231	MG763746	MG763745	/	/
Thaxteriella amazonensis	ATCC 42524	AY916458	AY787938	/	/
Thaxteriella inthanonensis	**MFLUCC11-0003 ✱**	**JN865211**	**JN865199**	**/**	**/**
Thaxteriellopsis lignicola	MFLUCC 16-0026	MH558768	MH558893	MH550960	MH551094
Thaxteriellopsis lignicola	MFLUCC 16-0024	MH558767	MH558892	MH550959	MH551093
Tubeufia abundata	**MFLUCC 17-2024 ✱**	**MH558769**	**MH558894**	**MH550961**	**MH551095**
Tubeufia aquatica	MFLUCC 15-0990	MT627687	MN913689	MT954399	MT878463

Tubeufia aquatica	MFLUCC 17-1794	MH558770	MH558895	MH550962	MH551096
Tubeufia bambusicola	**MFLUCC 17-1803 ✱**	**MH558771**	**MH558896**	**MH550963**	**MH551097**
Tubeufia brunnea	**MFLUCC 17-2022 ✱**	**MH558773**	**MH558898**	**MH550965**	**MH551099**
Tubeufia chiangmaiensis	MFLUCC 17-1801	MH558774	MH558899	MH550966	MH551100
Tubeufia cylindrothecia	MFLUCC 17-1792	MH558776	MH558901	MH550968	MH551102
Tubeufia dictyospora	**MFLUCC 17-1805 ✱**	**MH558778**	**MH558903**	**MH550970**	**MH551104**
Tubeufia fangchengensis	**MFLUCC 17-0047 ✱**	**MH558783**	**MH558908**	**MH550975**	**MH551109**
Tubeufia filiformis	MFLUCC 16-1135	KY092416	KY092411	KY117032	MF535285
Tubeufia hechiensis	**MFLUCC 17-0052 ✱**	**MH558785**	**MH558910**	**MH550978**	**MH551112**
Tubeufia krabiensis	MFLUCC 16-0324	MH275090	MH260324	MH412787	MH412761
Tubeufia latispora	**MFLUCC 16-0027 ✱**	**KY092417**	**KY092412**	**KY117033**	**MH551119**
Tubeufia laxispora	MFLUCC 17-2023	MH558794	MH558919	MH550987	MH551121
Tubeufia machaerinae	**MFLUCC 17-0055 ✱**	**MH558795**	**MH558920**	**MH550988**	**MH551122**
Tubeufia mackenziei	**MFLUCC 16-0222 ✱**	**KY092415**	**KY092410**	**KY117031**	**MF535288**
Tubeufia parvispora	MFLUCC 17-2009	MH558798	MH558923	MH550991	MH551125
Tubeufia parvispora	MFLUCC 17-1992	MH558796	MH558921	MH550989	MH551123
Tubeufia roseohelicospora	MFLUCC 17-1797	MH558800	MH558925	MH550993	MH551127
Tubeufia tectonae	**MFLUCC 17-1985 ✱**	**MH558810**	**MH558934**	**MH551003**	**MH551137**
Tubeufia tratensis	**MFLUCC 17-1993 ✱**	**NR_160398**	**MH558935**	**MH551004**	**MH551138**
Tubeufiaceae sp.	UBC F14999	AY916456	AY856870	/	/

Table S3. The names, isolate numbers, and corresponding GenBank accession numbers of the taxa used in Figure 5. The taxa produced in this study are indicated in red, and the type strains are indicated in bold with "✱"

Species	Strain	GenBank accession No.	
		ITS	LSU
Brunneocarpos banksiae	**CPC 29841 ✱**	**NR_147648**	**NG_066277**
Caliciopsis indica	**GUFCC 4947 ✱**	**GQ259981**	**GQ259980**
Chaenothecopsis consociata	Tibell 22472	AY795851	DQ008999
Chaenothecopsis debilis	Tibell 16643	AY795852	AY795991
Chaenothecopsis diabolica	**H:Tuovila 06-035 ✱**	**JX119109**	**JX119118**
Chaenothecopsis dolichocephala	**Tibell 19281 ✱**	**AY795854**	**AY795993**
Chaenothecopsis epithallina	Tibell 22705	AY795855	/
Chaenothecopsis fennica	Tibell 16024	AY795857	AY795995
Chaenothecopsis golubkovae	**Titov 6707 ✱**	**AY795859**	**AY795996**
Chaenothecopsis haematopus	CBS:173.95	MH862507	MH874148
Chaenothecopsis haematopus	16625	AY795861	AY795997
Chaenothecopsis hongheensis	HKAS 122672	OQ379009	OQ379420
Chaenothecopsis hongheensis	**HKAS 122673 ✱**	**OQ379010**	**OQ379421**
Chaenothecopsis khayensis	**H:JR 04G058 ✱**	**JX122785**	**HQ172895**
Chaenothecopsis matai	PDD 110746	OQ308931	OQ308874
Chaenothecopsis matai	**PDD 110749 ✱**	**OQ308932**	**OQ308875**
Chaenothecopsis montana	**H:Tuovila 07-086 ✱**	**JX119105**	**JX119114**
Chaenothecopsis nana	**Tibell 22473 ✱**	**AY795862**	/
Chaenothecopsis neocaledonica	**Rikkinen 010179 ✱**	**KF815196**	**KF815197**
Chaenothecopsis nigripunctata	**H:Tuovila 06-013 ✱**	**JX119103**	**JX119112**
Chaenothecopsis nodosa	PDD 110743	OQ308933	OQ308876
Chaenothecopsis nodosa	**PDD 110745 ✱**	**OQ308934**	**OQ308877**
Chaenothecopsis novae-zelandiae	PDD 110742	OQ308935	OQ308878
Chaenothecopsis novae-zelandiae	**PDD 110744 ✱**	**OQ308936**	**OQ308879**
Chaenothecopsis pallida	**H:JR 10652 ✱**	**JX122779**	**JX122781**
Chaenothecopsis pusilla	Tibell 1658	/	DQ009000
Chaenothecopsis pusiola	H:Tuovila 09-047	JX119106	JX119115
Chaenothecopsis quintralis	**BCRU:05233 ✱**	/	**JQ267741**
Chaenothecopsis resinicola	**Tibell 19234 ✱**	**AY795867**	/
Chaenothecopsis resinophila	**H:JR 000424 ✱**	**JX122780**	**JX122782**
Chaenothecopsis schefflerae	Rikkinen 13183	KY499965	KY499967
Chaenothecopsis sitchensis	**H:Tuovila 06-033 ✱**	**JX119102**	**JX119111**
Chaenothecopsis sp. 1	Tuovila 09-052	JX119110	JX119119
Chaenothecopsis sp. 2	Tuovila 08-004	KC590480	KC590485
Chaenothecopsis tsugae	H:JR07005B	JX119104	JX119113
Chaenothecopsis vainioana	H:Tuovila 09-066	JX119107	JX119116
Chaenothecopsis viridireagens	Tibell 22803	AY795872	DQ013257

Mycocalicium albonigrum	Tibell 19038	AF223966	AY796001
Mycocalicium hyaloparvicellulum	**MFLUCC 14-0169 ✱**	**KR920004**	**KR920005**
Mycocalicium sequoiae	**Rikkinen 92055 ✱**	**/**	**AY796002**
Mycocalicium sp. 2	Tuovila 09-131	KC590482	KC590487
Mycocalicium sp.1	Tibell 17604	AF243133	/
Mycocalicium subtile	Tibell 21020	AF225445	AY796003
Mycocalicium subtile	JR6450	OQ308930	OQ308873
Phaeocalicium interruptum	Tibell 23044	AY795873	/
Phaeocalicium polyporaeum	ZW-Geo60-Clark	AY789363	AY789362
Phaeocalicium populneum	Tibell 19286	AY795874	AY796009
Phaeocalicium praecedens	Tuovila 09-240	KC590481	KC590486
Phaeocalicium sp.	Tuovila 10-017	KC590483	KC590484
Phaeocalicium triseptatum	**L280 ✱**	**KJ871615**	**/**
Rhopalophora clavispora	CBS 129.74	KX537751	MH872573
Sphinctrina intermedia	**L281 ✱**	**KJ865747**	**/**
Sphinctrina leucopoda	Kalb 33829	AY795875	AY796006
Sphinctrina turbinata	Tibell 22478	AY795876	AY796007
Sphinctrina turbinata	Tibell 23093	AY795877	DQ009001
Stenocybe pullatula	Tibell 17117	AY795878	AY796008

Table S4. The names, isolate numbers, and corresponding GenBank accession numbers of the taxa used in Figure 7. The taxa produced in this study are indicated in red, and the type strains are indicated in bold with "✱".

Species name	Strain	GenBank accession No.	
		ITS	*tub2*
Camarops amorpha	SMH1450	AY780054	AY780093
Camarops petersii	JM1655	AY346265	AY780094
Camarops tubulina	SMH4614	AY346266	AY780095
Camarops ustulinoides	SMH1988	AY346267	AY780096
Chaetosphaeria biapiculata	**SMH 3074 ✱**	**AF466065**	**AF466026**
Chaetosphaeria cubensis	SMH 3258	AF466067	AF466028
Chaetosphaeria decastyla	SMH 2629	AF466068	AF466029
Chaetosphaeria hebetiseta	SMH 2729	AF466069	AF466030
Chaetosphaeria innumera	SMH 2748	AY017375	AF466018
Chaetosphaeria lignomollis	SMH 3015	AF466073	AF466034
Chaetosphaeria longiseta	SMH 3854	AF279417	AF466036
Chaetosphaeria luquillensis	**SMH 2973 ✱**	**AF466074**	**AF466037**
Chaetosphaeria minuta	SMH 3396	AF466075	AF466038
Chaetosphaeria ovoidea	SMH2605	AF064641	AF466057
Chaetosphaeria pygmaea	UPSC 2523	AF466077	AF466040

Coniochaetidium savoryi	TRTC 51980	AY346276	AY780114
Diaporthe phaseolorum	FAU458	AY346279	AY780115
Echinosphaeria canescens	JHC97-006	KF765604	KF765622
Echinosphaeria canescens	SMH4666	KF765605	KF765623
Echinosphaeria canescens	SMH4791	AY436403	/
Echinosphaeria canescens	TL5730	AY436404	KF765624
Eutypa sp.	SMH3580	AY346280	AY780117
Fusarium ambrosium	SMH1999	AY780077	AY780137
Helminthosphaeria carpathica	SMH3903	KF765606	KF765625
Helminthosphaeria cf. stuppea	JF04120	KF765611	KF765632
Helminthosphaeria* cf. *stuppea	**TL11998 ✱**	**KF765612**	**KF765633**
Helminthosphaeria clavariarum	**ANM Acc.17 ✱**	/	**KF765626**
Helminthosphaeria corticiorum	JF04225	KF765607	KF765627
Helminthosphaeria hispidissima	**ILLS00121145 ✱**	**MN447129**	/
Helminthosphaeria hyphodermiae	SMH4192	KF765608	KF765628
Helminthosphaeria ludens	**JF04126 ✱**	/	**KF765629**
Helminthosphaeria mammillata	ANM986	KF765609	KF765630
Helminthosphaeria odontiae	ANM928	KF765610	KF765631
Helminthosphaeria tomaculum	**SMH2485 ✱**	**KF765613**	**KF765634**
Helminthosphaeria triseptata	**JF02048 ✱**	/	**KF765635**
Helminthosphaeria triseptata	JF04015	KF765614	KF765636
Hilberina caudata	**SMH1542 ✱**	**KF765615**	**KF765637**
Hilberina hongheensis	**HKAS 122677 ✱**	**OQ379011**	**OQ379003**
Hilberina hongheensis	HKAS 122678	OQ379012	OQ379004
Hilberina munkii	SMH1531	KF765616	KF765638
Hilberina punctata	**SMH4825 ✱**	/	**KF765639**
Hilberina robusta	**SMH3054 ✱**	/	**KF765640**
Hilberina sphagnorum	**Buck49156 ✱**	**KF765617**	**KF765641**
Lasiosphaeria ovina	**SMH1538 ✱**	**AF064643**	**AF466046**
Melanochaeta aotearoae	SMH 3551	AF466082	AF466048
Melanochaeta hemipsila	**SMH2125 ✱**	**AY346292**	**AF466049**
Neurospora crassa	MUCL 19026	AF286411	M13630
Poroconiochaeta discoidea	**SANK 12878 ✱**	**AY346297**	**AY780134**
Ruzenia spermoides	ANM163	KF765618	KF765642
Ruzenia spermoides	SMH4606	AY436422	KF765644
Ruzenia spermoides	SMH4655	KF765619	KF765645
Synaptospora plumbea	ANM963	KF765620	KF765646
Synaptospora plumbea	SMH3962	KF765621	KF765647
Valsa ceratosperma	AR3426	AF408387	AY780144
Valsonectria pulchella	**SMH1193 ✱**	**AY346304**	**AY780145**

Table S5. The names, isolate numbers, and corresponding GenBank accession numbers of the taxa used in Figure 9. The taxa produced in this study are indicated in red, and the type strains are indicated in bold with "✱"

Species	Strain	GenBank accession No.		
		ITS	LSU	*rpb2*
Amphibambusa bambusicola	**MFLUCC 11-0617 ✱**	**KP744433**	**KP744474**	**/**
Amphibambusa hongheensis	**KUMCC 20-0334**	**MW892972**	**MW892970**	**/**
Arecophila bambusae	HKUCC 4794	/	AF452038	/
Arecophila muroiana	GZUCC0122	MT742127	MT742134	/
Biscogniauxia capnodes	YMJ 138	EF026131	/	JX507779
Biscogniauxia nummularia	**MUCL 51395 ✱**	**JX658444**	**KT281894**	**KY624236**
Biscogniauxia petrensis	LC5697	KU746669	KU746715	/
Cainia graminis	CBS 136.62	MH858123	MH869701	/
Cainia sp.	LSU0560	MT000421	MT000513	/
Delonicicola siamense	**MFLUCC 15–0670 ✱**	**MF167586**	**MF158345**	**MF158346**
Delonicicola siamense	KUMCC 21-0459	OQ379013	OQ379424	OQ378992
Discula destructiva	**ATCC 76230**	**DQ323528**	**AF362568**	**/**
Graphostroma platystoma	AFTOL-ID 1249	/	DQ836906	DQ836893
Kretzschmaria deusta	CBS163.93	KC477237	KY610458	/
Liberomyces macrosporus	**CCF 4028 ✱**	**NR_154605**	**/**	**FR715509**
Liberomyces pistaciae	CPC 33866	MH797616	/	MH797684
Liberomyces saliciphilus	H041	FR715510	/	FR715507
Obolarina dryophila	8401 = H86	/	GQ428313	FR715505
Podosordaria tulasnei	CBS 128.80	KT281902	KT281897	/
Seynesia erumpens	SMH 1291	/	AF279410	AY641073
Xylaria hypoxylon	ATCC 42768	AY327477	AY327480	/

Table S6. The names, isolate numbers, and corresponding GenBank accession numbers of the taxa used in Figure 11. The taxa produced in this study are indicated in red, and the type strains are indicated in bold with "✱"

Species	Strain	GenBank accession No.		
		LSU	*tef1-a*	*rpb2*
Acanthonitschkea argentinensis	SMH1395	AY695259	FJ969042	FJ968943
Acanthonitschkea tristis	SMH4723	FJ968949	FJ969043	/
Bertia gigantospora	**GKM1136 ✱**	**/**	**FJ969008**	**FJ968937**
Bertia orbis	**GKM1271 ✱**	**FJ968955**	**FJ969009**	**/**
Chaetosphaerella phaeostroma	SMH4585	AY346274	FJ969003	/
Chaetosphaerella phaeostroma	SMH4257	AY695264	FJ969004	FJ968940
Coronophora gregaria	ANM1555	/	FJ969007	FJ968938
Coronophorella chaetomioides	**GKM1099 ✱**	**FJ968969**	**FJ969034**	**FJ968922**
Cryptosphaerella celata	**GKM1231 ✱**	**FJ968975**	**FJ969035**	**FJ968929**
Cryptosphaerella costaricensis	**MO2111 ✱**	**FJ968971**	**FJ969028**	**/**
Cryptosphaerella cylindriformis	GKM1042	FJ968973	FJ969032	FJ968918
Cryptosphaerella cylindriformis	GKM1187	GQ217531	FJ969033	FJ968925
Cryptosphaerella cylindriformis	**GKM434N ✱**	**FJ968972**	**FJ969031**	**FJ968934**
Cryptosphaerella elliptica	**SMH4722 ✱**	**FJ968974**	**FJ969029**	**FJ968944**
Cryptosphaerella globosa	**GKM471N ✱**	**FJ968977**	**FJ969036**	**FJ968935**
Cryptosphaerella malindensis	**GKM1150 ✱**	**FJ968970**	**FJ969027**	**FJ968923**
Euacanthe foveolata	GKM1221	FJ968978	FJ969026	FJ968927
Fracchiaea broomeana	GKM1071	/	FJ969040	FJ968919
Fracchiaea broomeana	SMH347	FJ968979	FJ969041	FJ968947
Fracchiaea broomeana	SMH2809	AY695268	FJ969039	FJ968942

Fracchiaea lunata	GKM1089	/	FJ969005	FJ968921
Fracchiaea myricoides	**IFRD 9201 ✱**	**KX856174**	/	/
Fracchiaea myricoides	HKAS 122671	OQ379425	OQ378999	OQ378993
Neofracchiaea callista	SMH2689	AY695269	FJ969020	FJ968941
Nitschkia menicoidea	SMH1523	AY695270	/	/
Nitschkia tetraspora	SMH4787	FJ968984	FJ969010	/
Nitschkia tetraspora	GKML213N	FJ968985	/	/
Nitschkia tetraspora	SMH2469.2	FJ968986	/	/
Scortechinia acanthostroma	SMH1164	FJ968989	FJ969014	FJ968924
Scortechinia acanthostroma	SMH1143	FJ968988	FJ969012	FJ968948
Tympanopsis confertula	GKM1242	FJ968997	FJ969023	FJ968930
Tympanopsis confertula	ANM1567	FJ969001	FJ969025	FJ968939
Tympanopsis confertula	SMH4841	FJ968998	FJ969024	FJ968946
Tympanopsis uniseriata	GKM1203	FJ968999	FJ969016	FJ968926
Tympanopsis uniseriata	GKM1228	FJ969000	FJ969017	FJ968928

Table S7. The names, isolate numbers, and corresponding GenBank accession numbers of the taxa used in Figure 13. The taxa produced in this study are indicated in red, and the type strains are indicated in bold with "✱"

Species name	Strain	GenBank accession No.			
		ITS	LSU	SSU	*tef1-a*
Acrocordia subglobosa	HTL940	/	JN887392	JN887373	JN887417
Anisomeridium cf. *willeyanum*	MPN549	/	JN887393	/	/
Anisomeridium phaeospermum	MPN539	/	JN887394	JN887374	/
Anisomeridium sp.	MPN533	/	JN887395	JN887375	JN887419
Anisomeridium sp.	MPN540	/	JN887397	JN887377	JN887420
Anisomeridium sp.	MPN534	/	JN887396	JN887376	/
Anisomeridium sp.	MPN542	/	JN887398	JN887378	/
Anisomeridium ubianum	MPN94	/	GU327709	JN887379	/
Elsinoe centrolobii	**CBS 222.50 ✱**	**NR_148132**	**KX886969**	**NG_062717**	**DQ677934**
Elsinoe phaseoli	**CBS 165.31 ✱**	**NR_148161**	**DQ678095**	**NG_062718**	**DQ677935**
Eriomyces heveae	**MFLUCC 17-2232 ✱**	**NR_169673**	**MH109524**	**/**	**/**
Heleiosa barbatula	JK 5548I	/	GU479787	GU479753	/
Heleiosa barbatula	HKAS 122668	OQ379015	OQ379426	OQ372923	OQ379000
Italiofungus phillyreae	CPC 35566	MT223804	MT223899	/	/
Megalotremis verrucosa	Lucking 26316	/	GU327718	JN887383	/
Myriangium hispanicum	CBS 247.33	KX887304	GU301854	GU296180	GU349055
Neoheleiosa lincangensis	HKAS 111911	MW424766	MW424781	MW424796	MW430102
Neoheleiosa lincangensis	HKAS 111912	MW424765	MW424780	MW424795	MW430101
Neoheleiosa lincangensis	HKAS 111913	MW424767	MW424782	MW424797	MW430103
Neoheleiosa lincangensis	**HKAS 111914 ✱**	**MW424764**	**MW424779**	**MW424794**	**MW430100**

Pseudopassalora gouriqua	**CBS 101954 ✱**	**NR_160207**	**NG_067272**	**/**	**/**
Pseudopassalora gouriqua	CPC 1811	/	JN712565	/	/
Trypetheliopsis kalbii	MPN243	/	JN887406	JN887391	/

Table S8. The names, isolate numbers, and corresponding GenBank accession numbers of the taxa used in Figure 15. The taxa produced in this study are indicated in red, and the type strains are indicated in bold with "✱"

Species	Strain	GenBank Accession No.			
		ITS	LSU	*tef1-a*	*rpb2*
Anteaglonium globosum	**ANM925.2 ✱**	**/**	**GQ221879**	**GQ221925**	**/**
Anteaglonium parvulum	**MFLUCC 14-0821 ✱**	**/**	**KU922915**	**KU922921**	**/**
Hermatomyces amphisporus	ILLS 82999	LR812667	/	LR812661	LR812672
Hermatomyces amphisporus	ILLS 82998	LR812666	/	LR812660	LR812671
Hermatomyces amphisporus	ILLS 82997	LR812665	/	LR812659	LR812670
Hermatomyces amphisporus	ILLS 82991	LR812662	/	LR812657	LR812668
Hermatomyces amphisporus	ILLS 82994	LR812663	/	LR812658	LR812669
Hermatomyces bauhiniae	**MFLU 18-1381 ✱**	**MK443382**	**MK443378**	**MK443384**	**MK443386**
Hermatomyces sphaericus (*H. biconisporus*)	KUMCC 17-0183	MH275063	MH260296	/	/
Hermatomyces bifurcatus	**CCF 5900 ✱**	**LS398263**	**LS398263**	**LS398417**	**LS398344**
Hermatomyces bifurcatus	CCF 5899	LS398262	LS398262	LS398416	LS398343
Hermatomyces krabiensis (*H.*	MFLUCC 16-2819	/	KY559394	/	/

chiangmaiensis)					
Hermatomyces sphaericus (*H. chromolaenae*)	MFLUCC 16-2817	/	KY559393	/	/
Hermatomyces clematidis	**MFLUCC 17-2085 ✱**	**MT310603**	**MT214556**	**MT394735**	**MT394684**
Hermatomyces constrictus	**CCF 5904 ✱**	**LS398264**	**LS398264**	**LS398418**	**LS398345**
Hermatomyces indicus	MFLUCC 14-1145	KU144922	KU764694	KU872756	KU712490
Hermatomyces indicus	MFLUCC 14-1144	KU144921	KU764693	KU872755	KU712489
Hermatomyces indicus	**MFLUCC 14-1143 ✱**	**KU144920**	**KU764692**	**KU872754**	**KU712488**
Hermatomyces indicus	MFLUCC 21-0453	OQ379016	OQ379427	OQ379001	OQ378994
Hermatomyces iriomotensis	**KT2016-1 ✱**	**LC194483**	**LC194367**	**LC194394**	**LC194449**
Hermatomyces krabiensis	**MFLUCC 16-0249 ✱**	**KX525750**	**KX525742**	**KX525758**	**KX525754**
Hermatomyces megasporus	CCF 5897	LS398265	/	LS398419	LS398346
Hermatomyces megasporus	**CCF 5898 ✱**	**LS398266**	**LS398266**	**LS398420**	**/**
Hermatomyces nabanheensis	**KUMCC 16-0149 ✱**	**KY766058**	**KY766059**	**KY766061**	**/**
Hermatomyces sphaericus (*H. pandanicola*)	MFLUCC:160251	KX525751	KX525743	KX525759	KX525755
Hermatomyces reticulatus	CCF 5893	LS398267	LS398267	LS398421	LS398347
Hermatomyces reticulatus	CCF 5905	LS398268	LS398269	LS398422	LS398348
***Hermatomyces reticulatus* (*H. subiculosus*)**	**MFLUCC 15-0843 ✱**	**KX259521**	**KX259523**	**KX259527**	**KX259529**
Hermatomyces sphaericus (*H. saikhuensis*)	MFLUCC 16-0266	KX525748	KX525740	KX525756	KX525752
Hermatomyces sphaericus (*H. saikhuensis*)	MFLUCC 16-0267	KX525749	KX525741	KX525757	KX525753
Hermatomyces sphaericoides	CCF 5907	LS398272	/	LS398426	/
Hermatomyces sphaericoides	**CCF 5908 ✱**	**LS398273**	**LS398273**	**LS398427**	**LS398352**

Hermatomyces sphaericoides	KZP 470	LS398274	/	LS398428	LS398353
Hermatomyces sphaericoides	CCF 5895	LS398270	LS398270	LS398424	LS398350
Hermatomyces sphaericoides	CCF 5896	LS398271	/	LS398425	LS398351
Hermatomyces sphaericus	**MFLUCC 17-0915 ✱**	**/**	**/**	**MK360058**	**MK434868**
Hermatomyces sphaericus	CCF 5916	LS398284	LS398284	LS398433	LS398358
Hermatomyces sphaericus	CCF 5894	LS398277	LS398277	LS398429	LS398354
Hermatomyces sphaericus	KZP462	LS398287	LS398287	LS398434	LS398359
Hermatomyces sphaericus	CCF 5914	LS398283	LS398283	LS398432	LS398357
Hermatomyces sphaericus	CCF 5911	LS398281	LS398281	LS398431	LS398356
Hermatomyces sphaericus	CCF5901	LS398278	/	LS398430	LS398355
Hermatomyces sphaericus (*H. tectonae*)	MFLUCC 14-1140	KU144917	KU764695	KU872757	KU712486
Hermatomyces sphaericus (*H. tectonae*)	MFLUCC 14-1141	KU144918	KU764696	KU872758	/
Hermatomyces sphaericus (*H. tectonae*)	MFLUCC 14-1142	KU144919	KU764697	/	KU712487
Hermatomyces thailandicus	MFLUCC 20-0114	MT883355	MT883353	/	/
Hermatomyces trangensis	BCC 80742	KY790599	KY790601	KY790607	KY790605
Hermatomyces trangensis	**BCC 80741 ✱**	**KY790598**	**KY790600**	**KY790606**	**KY790604**
Hermatomyces tucumanensis	CCF 5915	LS398290	LS398290	LS398437	LS398362
Hermatomyces tucumanensis	CCF 5913	LS398289	LS398289	LS398436	LS398361
Hermatomyces tucumanensis	CCF 5912	LS398288	LS398288	LS398435	LS398360
Hermatomyces verrucosus	CCF 5892	LS398291	LS398291	LS398438	LS398363
Hermatomyces verrucosus	**CCF 5903 ✱**	**LS398292**	**LS398292**	**LS398439**	**LS398364**
